# Brain region-specific microglial and astrocytic activation in response to systemic lipopolysaccharides exposure

**DOI:** 10.3389/fnagi.2022.910988

**Published:** 2022-08-26

**Authors:** Edoardo Brandi, Laura Torres-Garcia, Alexander Svanbergsson, Caroline Haikal, Di Liu, Wen Li, Jia-Yi Li

**Affiliations:** ^1^Neural Plasticity and Repair Unit, Department of Experimental Medical Science, Lund University, Lund, Sweden; ^2^Health Sciences Institute, China Medical University, Shenyang, China

**Keywords:** microglia, neuroinflammation, lipopolysaccharide (LPS), CX3CR1, substantia nigra (SN), astrocyte, dopaminergic (DA) neuron

## Abstract

Microglia cells are the macrophage population within the central nervous system, which acts as the first line of the immune defense. These cells present a high level of heterogeneity among different brain regions regarding morphology, cell density, transcriptomes, and expression of different inflammatory mediators. This region-specific heterogeneity may lead to different neuroinflammatory responses, influencing the regional involvement in several neurodegenerative diseases. In this study, we aimed to evaluate microglial response in 16 brain regions. We compared different aspects of the microglial response, such as the extension of their morphological changes, sensitivity, and ability to convert an acute inflammatory response to a chronic one. Then, we investigated the synaptic alterations followed by acute and chronic inflammation in substantia nigra. Moreover, we estimated the effect of partial ablation of fractalkine CX3C receptor 1 (CX3CR1) on microglial response. In the end, we briefly investigated astrocytic heterogeneity and activation. To evaluate microglial response in different brain regions and under the same stimulus, we induced a systemic inflammatory reaction through a single intraperitoneal (i.p.) injection of lipopolysaccharides (LPS). We performed our study using C57BL6 and CX3CR1^+/GFP^ mice to investigate microglial response in different regions and the impact of CX3CR1 partial ablation. We conducted a topographic study quantifying microglia alterations in 16 brain regions through immunohistochemical examination and computational image analysis. Assessing Iba1-immunopositive profiles and the density of the microglia cells, we have observed significant differences in region-specific responses of microglia populations in all parameters considered. Our results underline the peculiar microglial inflammation in the substantia nigra pars reticulata (SNpr). Here and in concomitance with the acute inflammatory response, we observed a transient decrease of dopaminergic dendrites and an alteration of the striato-nigral projections. Additionally, we found a significant decrease in microglia response and the absence of chronic inflammation in CX3CR1^+/GFP^ mice compared to the wild-type ones, suggesting the CX3C axis as a possible pharmacological target against neuroinflammation induced by an increase of systemic tumor necrosis factor-alpha (TNFα) or/and LPS. Finally, we investigated astrocytic heterogeneity in this model. We observed different distribution and morphology of GFAP-positive astrocytes, a heterogeneous response under inflammatory conditions, and a decrease in their activation in CX3CR1 partially ablated mice compared with C57BL6 mice. Altogether, our data confirm that microglia and astrocytes heterogeneity lead to a region-specific inflammatory response in presence of a systemic TNFα or/and LPS treatment.

## Introduction

Microglia cells represent the first line of the immune defense within the central nervous system (CNS), and they are widely present in the brain. Despite their ubiquitous distribution, these cells differ among different brain regions. Lawson et al. (1990) showed that microglia populations appeared of high heterogeneity in their morphology and cell density. Other studies reported differential expression of crucial inflammatory mediators, such as tumor necrosis factor-alpha (TNFα), interleukin 1-beta (IL1β), and fractalkine chemokine axis CX3C, among microglia cells of different brain regions (de Haas et al., 2008; Grabert et al., 2016; De Biase et al., 2017; Ayata et al., 2018; Abellanas et al., 2019; Masuda et al., 2020). Interestingly, the marked difference in cell density and morphology that occurs between microglia cells in the substantia nigra pars reticulata (SNpr) compared to the ones in SN pars compacta (SNpc) and ventral tegmental area (VTA). These neighboring regions contain the highest and lowest microglia densities in the whole CNS and evident morphological differences in their branches and lysosomal system (De Biase et al., 2017). De Biase et al. (2017) performed a whole transcriptome RNA-sequencing data analysis, showing that microglia populations in SN, VTA, Nucleus Accumbens (NAc), and cortex share only 48% of their gene expression profile in a steady-state condition. Abellanas et al. (2019) examined microglia in the midbrain, striatum, cortex, and hippocampus, observing variation in the distribution of critical inflammatory components such as toll-like receptor 4 (TLR4) and major histocompatibility complex I or II (MHC I-II). They showed that microglial cells in the midbrain contained higher levels of these components.

A useful model to study neuroinflammation is represented by the one obtained through lipopolysaccharide (LPS) administration. This compound is a component of the extracellular membrane of gram-negative bacteria that can induce a systemic inflammatory response when administered intraperitoneally (i.p.). In turn, this systemic inflammation can affect the brain mainly *via* TNFα signaling, inducing the activation of microglia cells (Rietschel et al., 1994; Qin et al., 2007). In some regions, such as circumventricular organs (CVO) in the hypothalamus, the lack of blood-brain barrier (BBB) exposes the tissue to the direct stimulation from LPS, mediated by TLR4 (Furube et al., 2018; Vargas-Caraveo et al., 2020). Studies also showed that LPS could pass the BBB through lipoprotein-mediated transport but in low amounts (Vargas-Caraveo et al., 2017). In TNFα -depleted mice, the general neuroinflammatory response was abolished, indicating that TNFα is crucial for the inflammation observed in the brain after systemic LPS administration (Qin et al., 2007). Studies have further described that the inflammatory response within the brain could be converted from an acute reaction to a chronic response, which induces subsequent progressive dopaminergic neurodegeneration in SNpc (Qin et al., 2007; Liu et al., 2008; Zheng et al., 2013). However, other studies failed to show such chronic inflammation in the cerebral cortex 2 weeks after LPS administration (Benusa et al., 2017). Whether these discrepancies depend on a distinct inflammatory response between several brain regions, differences in the treatments or various forms of LPS used in several studies is still unclear. Most of the works that investigated chronic inflammation have been focused on a limited number of brain regions. The lack of comparisons about the uniformity of inflammatory response in the whole brain leaves this question open. Recently, Zhao et al. (2020) showed that chronic inflammation could be driven by an IL1β-dependent mechanism, while other studies have shown that IL1β expression differs between brain regions after systemic LPS administration (Silverman et al., 2014; Abellanas et al., 2019). Consequently, these data suggest that chronic inflammation may differ among several brain regions. Moreover, it was shown that caspase-1, responsible for IL1β maturation, is also able to induce truncation of α-synuclein protein, increasing its ability to get aggregated (Ulusoy et al., 2010; Games et al., 2014; Tran et al., 2014; Wang et al., 2016; Rai et al., 2021). Consequently, the different expression level of IL1β, reflecting the activity of caspase-1, could also influence the spatial-distribution of Lewy bodies, one of the main characteristic of synucleinopathies.

In the present study, we have investigated whether microglia heterogeneity leads to different inflammatory responses among 16 brain regions after exposure to the same pro-inflammatory stimulus. Using an LPS model obtained with a single i.p. injection in C57BL6 mice, we observed brain regional-specific responses regarding several microglia activation parameters, including (1) alterations in morphology and changes in lysosomal system, reflecting the extent of microglia activation, (2) their sensitivity to systemic TNFα/LPS, and (3) their ability to preserve the activated state chronically 1 month after treatment. Interestingly, microglia cells in SNpr exhibited the most robust inflammatory response, were particularly sensitive to systemic TNFα/LPS, and maintained an activated status chronically. This inflammatory condition transiently decreased tyrosine hydroxylase (TH) positive profiles in SNpr. Moreover, we compared microglia activation in CX3CR1^+/GFP^ and C57BL6 mice after systemic LPS administration. We observed lower microglia activation in CX3CR1^+/GFP^ 24 h after treatment, and the absence of chronic microglia activation 1-month post-LPS administration.

Lastly, we explored whether astrocytes present a regional-specific distribution and phenotypes. Our data revealed a high heterogeneity of this cell population and a region-specific inflammatory response under inflammatory conditions. Moreover, we observed a lower astrocytic activation in CX3CR1^+/GFP^ mice compared to C57BL6 mice, confirming that CX3CR axis could represent an important pharmacological target to decrease the inflammatory reaction within the brain.

## Materials and methods

### Animals

Adult male 6-month-old C57BL6 were purchased from Janvier Labs (Saint Berthevin Cedex, France), and they were acclimatized for 19 days before the experiments. Adult male 6-months-old CX3CR1^+/GFP^ were bred *in loco*. Mice were housed in a controlled environment with the temperature at 21°C, controlled humidity, 12 h light/dark cycle, cages enriched by *stimuli*, fed *ad libitum* with rodent pellet diet, and free access to water. All animals included in this work and the procedures performed are approved by the Malmö/Lund Animal Ethical Committee (Dnr 5.8.18-09454/2021).

### Lipopolysaccharides treatments and samples collection

Mice received a single i.p. injection of LPS (clone O55:B5, Sigma-Aldrich, St. Louis, MO, United States) or vehicle alone and were sacrificed 1- or 30-days post-treatment. 5 mg/kg concentration of LPS was used to induce the acute and chronic response and 5, 0.5, 0.05, and 0.005 mg/kg doses were used for the dose-dependent response experiments. All experiments involved three animals in each group and were performed simultaneously. At the end of the experiments, mice were deeply anesthetized using pentobarbital and perfused with saline, followed by 4% paraformaldehyde (PFA). Brains were post-fixed in the same fixative for one overnight and then immersed in PBS with 30% sucrose until use.

### Immunostaining and microscopy

Brains were cut into 25 um thick sections using a microtome (Leica SM2010R, Microtome and Microscope, Leica Microsystems, Wetzlar, Germany) and stored at −20°C in an anti-freeze solution. Brain slices were stained for IIba1 antibody (1:1000, Nordic Biolabs, Wako Chemicals, Täby, Sweden, Cat. #019-19741), CD68 antibody (1:500, Bio-Rad, Hercules, CA, United States, Cat. #MCA1957), TH antibody (1:2000, Chemicon-Millipore, Burlington, MA, United States, Cat. #AB152 and #MAB358), Gephyrin antibody (1:250, Synaptic Systems, Göttingen, Germany, Cat. #147011C3), GFAP antibody (1:500, Dako, Santa Clara, CA, United States, Cat. #Z0334), in PBS with the serum (5–10%) from the animal species of the secondary antibodies and Triton-X 100. The images were acquired using Olympus Virtual Stage 120 with extended focus imaging setting (EFI) for scan images (20x and 10x magnifications), Olympus BX53 microscopes, Shinjuku, Tokyo, Japan (20x and 60x magnification), and LEICA Stellaris 8 Dive for confocal images (40x and 63x magnification). EFI setting consists of five optical sections with a 5 um increment between two consecutive optical sections. Z-stack confocal images consist of 30 optical sections, with 0.5 um increment between two consecutive optical sections, and their 3-dimensional projection was used for analyses.

### Quantitative image analyses

We examined 16 different brain regions: (1) Different representative nuclei, such as NAc, striatum, thalamus, hypothalamus, amygdala, VTA, SNpc, and SNpr; (2) Different regions of the cerebral cortex: primary motor and somatosensory cortex (PSM cortex), posterior parietal association areas (PPA cortex), primary visual area (visual cortex, PC), hippocampus, entorhinal cortex, posterior piriform cortex, and the simple lobule of cerebellum; (3) anterior corpus callosum was the only white matter structure considered. Regions of interest (ROIs) followed coordinates from Allen Brain Atlas, as shown in few examples in Supplementary Figure 1A.

We evaluate microglia activation considering changes in the percentage of the marked area and cell density. We performed image analyses using ImageJ software and different homemade macros adapted to our purpose. Iba 1 marked areas were detected using a set threshold able to distinguish cell soma and main branches (Supplementary Figure 1B). Cell density was measured using a threshold able to identify the soma of microglia cells. We applied several transformations and size filters to detect the cell soma specifically, as shown in Supplementary Figure 1C.

To quantify TH and Gephyrin, we used confocal images and ImageJ software with homemade macros and set thresholds. To assess GFAP and CD68 alterations in response to the exposure of LPS, we randomly selected 4–7 frames of 20x images per animal and analyzed them using ImageJ software and homemade macros. We quantified GFAP using a set threshold while we performed a semi-quantitative analysis of CD68 with variable thresholding.

### Statistical analysis

Statistical analyses were performed using Graph Pad Prism 9 as software. All graphed values are shown as means with standard deviations, and statistical details are described in the results and figure legends. Generally, statistical significance was assessed using a one-way ANOVA with Tukey or Sídák correction, two-way ANOVA with Tukey correction, and unpaired student *t*-test. Significances are partially summarized in the graphs, and more detailed information is provided in Supplementary Tables 1–6.

## Results

### Microglia heterogeneity in C57BL6 mice in naive condition

**FIGURE 1:**
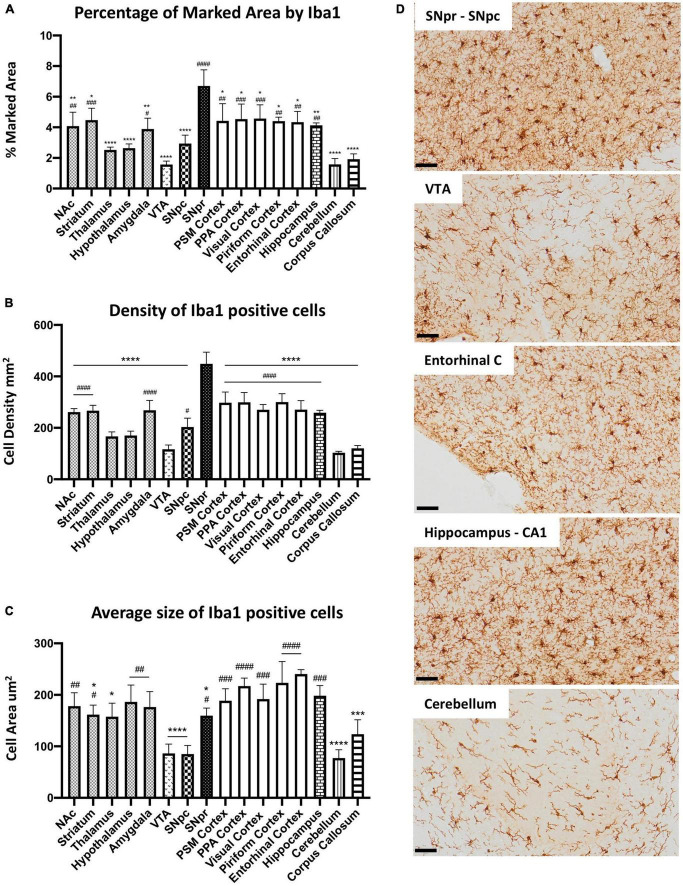
Microglial heterogeneity in C57BL6N mice. **(A)** Percentage of the marked area by Iba1 in vehicles C57BL6N mice. One-way ANOVA *F*_Brain Regions_ (15, 32) = 12.06, *p* < 0.0001, and *n* = 3 mice per region. Significance: (*) respect SNpr; (#) respect VTA. Other significances are summarized in Supplementary Table 1. **(B)** Iba 1 positive cell density per mm^2^. One-way ANOVA *F*_Brain Regions_ (15, 32) = 29.35, *p* < 0.0001, and *n* = 3 mice per region. Significance: (*) respect SNpr; (#) respect VTA. Other significances are summarized in Supplementary Table 2. **(C)** Average size of Iba1 positive cells. One-way ANOVA *F*_Brain Regions_ (15, 32) = 12.65, *p* < 0.0001, and *n* = 5 cells per region, and *n* = 3 mice per region. Significance: (*) respect Entorhinal cortex; (#) respect VTA and SNpc. Other significances are summarized in Supplementary Table 3. **(D)** Representative images of Iba 1 staining in SNpr, SNpc, VTA, entorhinal cortex, hippocampus (CA1), and cerebellum. Scale bar **(D)** = 50 um.

To study microglia heterogeneity in C57BL6 mice, we first characterized microglia cells across the different brain regions in control mice without LPS exposure. SNpr represented the brain region with the highest percentage of the marked area by Iba1 (Figures 1A,D). Comparatively, microglia coverage in the cerebellum, VTA, and corpus callosum was the lowest, followed by the hypothalamus, thalamus, and SNpc. The remaining nine regions showed similar microglia marked area. We observed a similar situation when we analyzed the cell density (Figures 1B,D). Then, we examined microglia size and morphology, considering their branch structures. For this purpose, we performed confocal microscopy imaging in Iba1 immuno-labeled sections. We conducted cell size analysis creating ROIs around single cells. This analysis (Figure 1C) showed that microglia cells in 12 regions present no significant difference in cell size. Interestingly, the size of microglia cells in the cerebellum, corpus callosum, VTA, and SNpc was significantly smaller than in other brain regions. After that, we performed a qualitative morphological analysis, considering the complexity of branch structure. We distinguished the branches between “simple” and “complex” based on the number of branches that derived from cell soma and by the presence of further ramifications on each branch. From this analysis, we observed the simplest branch structure in VTA, cerebellum, corpus callosum, SNpc, thalamus, and hypothalamus, while all other regions showed a complex structure (Supplementary Figure 2).

To further investigate microglial heterogeneity, we performed a qualitative analysis of the cluster of differentiation 68 (CD68), a microglial-specific transmembrane glycoprotein localized primarily on lysosomal and endosomal vesicles. From this analysis, we generally observed the same pattern as for Iba1. The heterogeneity between the different brain regions seems to depend mainly on differences in microglia density. However, CD68 positive profiles were more robust in SNpr compared with other regions, while the hypothalamus and VTA showed lower amounts. Interestingly, the CD68 positive profile seems more intense in the PSM cortex and cerebellum concerning all other regions (Supplementary Figure 3). These data showed that nine of the examined regions present a similar percentage of Iba1 marked area and cell density in physiological conditions, while the other seven appeared to be more heterogeneous. Nuclei exhibited higher variability than cortical regions. Morphologically, microglia cells were heterogeneous, and their size depended primarily on the complexity of their branch structure. Moreover, further differences could be present considering other microglial features such as the lysosomal or endosomal system.

### Brain region-specific microglia responses to intraperitoneal administration of lipopolysaccharides 5 mg/kg

**FIGURE 2:**
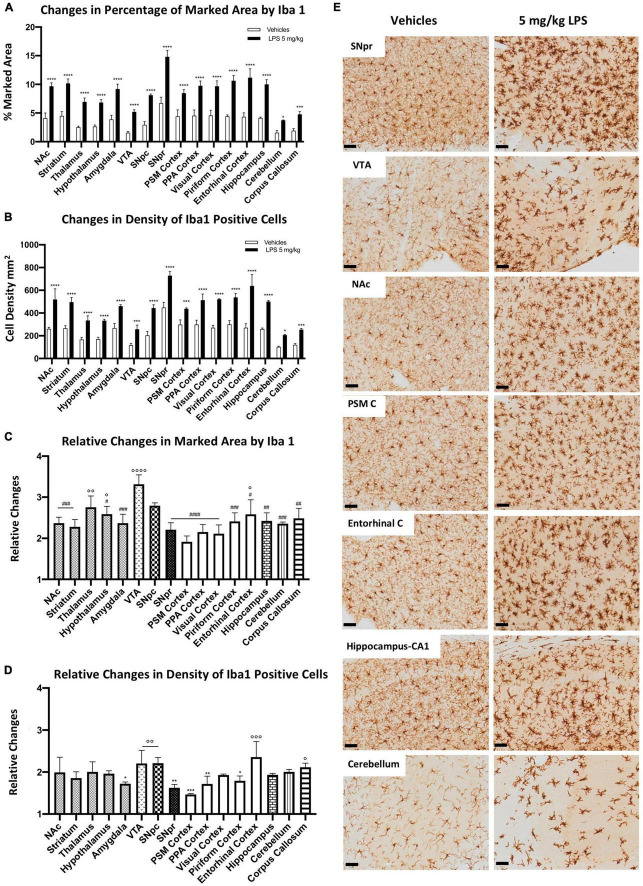
Heterogeneous microglial alterations 24 h after 5 mg/kg LPS administration. **(A)** Percentage of the marked area by Iba1 in vehicles (white columns) and LPS treated mice (black columns). Two-way ANOVA *F*_Brain Regions_ (15, 64) = 46.13, *p* < 0.0001. *F*_Treatment_ (1, 64) = 1127, *p* < 0.0001. *F*_Interaction_ (15, 64) = 6.081, *p* < 0.0001, and *n* = 3 mice per region. Significance: (*) respect relative vehicles. **(B)** Iba 1 positive cell density per mm^2^ in vehicles (white columns) and LPS treated mice (black columns). Two-way ANOVA *F*_Brain Regions_ (15, 64) = 56.84, *p* < 0.0001. *F*_Treatment_ (15, 64) = 786.7, *p* < 0.0001. *F*_Interaction_ (15, 64) = 5.138, *p* < 0.0001, and *n* = 3 mice per region. Significance: (*) respect relative vehicles. **(C)** Relative changes in the percentage of marked area by Iba1 between LPS and vehicle-treated mice. One-way ANOVA *F*_Brain Regions_ (15, 32) = 7.453, *p* < 0.0001, and *n* = 3 mice per region. Significance: (#) respect VTA; (°) respect PSM cortex. Other significances are summarized in Supplementary Table 4A. **(D)** Relative changes in density of Iba1 positive cells between LPS and vehicle-treated mice. One-way ANOVA *F*_Brain Regions_ (15, 32) = 4,890 *p* < 0.0001, and *n* = 3 mice per region. Significance: (*) respect the Entorhinal cortex; (°) respect the PSM cortex. Other significances are summarized in Supplementary Table 4B. **(E)** Representative images of Iba 1 staining in SNpr, VTA, NAc, PSM cortex, entorhinal cortex, hippocampus (CA1), and cerebellum. Scale bar = 50 um.

To evaluate whether microglia heterogeneity could contribute to different inflammatory responses among different brain regions, we compared microglia activation in mice treated systematically with either 5 mg/kg LPS or vehicle (PBS). We detected a significant increase of the marked area by the Iba1-positive profile (Figures 2A,E) and cell density (Figures 2B,E) in all brain regions upon LPS exposure. SNpr and the entorhinal cortex presented the most robust microglia activation. Comparatively, the activation of microglia cells in the VTA, cerebellum, and corpus callosum was the lowest, although the response was still statistically significant upon LPS treatment compared to the control mice. Although the extent of activation depends on the baseline level of the examined brain regions, these data still suggest a difference in inflammatory responses between various brain regions.

When we examined the fold changes between LPS-treated and control groups, we observed a 2–3-fold increase in microglia marked area (Figure 2C) and similar changes in microglia density (Figure 2D). The most significant increase in the microglia marked area appeared in the VTA. In contrast, the lowest changes were detected in the PSM cortex (Figure 2C). Concerning the relative changes in cell density (Figure 2D), the entorhinal cortex, VTA, and SNpc showed the highest differences, while the PSM and SNpr were the lowest. These differences in microglial activation were evident when comparing PSM and entorhinal cortices (Figure 2E). These regions present similar microglia marked area and density in control conditions. However, in LPS treated mice, microglia activation was more robust in the entorhinal cortex than PSM cortex.

In the end, we performed a semi-quantitative analysis of CD68 changes under LPS exposure to confirm a differential microglial response among different brain regions. We observed a general increase in all regions, which appeared dependent both on cell density increase and on intensity of CD68 immunoreactivity. The most evident change occurred in the SNpr, where the signal increased stronger than that in all other regions (Supplementary Figure 4). These data indicate differential microglial responses to LPS exposure among different brain regions.

### Brain region-specific microglia activation in response to different doses of lipopolysaccharides

**FIGURE 3:**
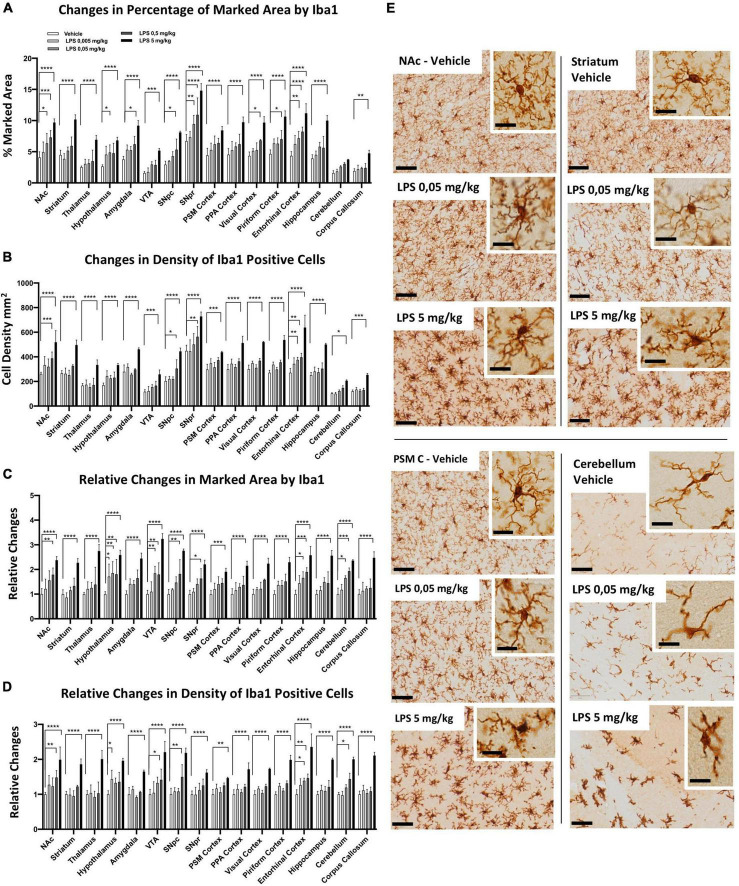
Regional specific microglia activation to different doses of LPS in C57BL6 mice. **(A)** Percentage of marked area by Iba1 in vehicles (white columns), LPS treated mice with 0.005 mg/kg (clear gray columns), 0.05 mg/kg (medium gray columns), 0,5 mg/kg (dark gray columns), 5 mg/kg (black columns). Two-way ANOVA. *F*_Brain Regions_ (15, 160) = 57.94, *p* < 0.0001. *F*_Treatment_ (4, 160) = 188,3, *p* < 0.0001. *F*_Interaction_ (60, 160) = 1.599, *p* < 0.0110, and *n* = 3 mice per region. Significance: respect relative vehicles. **(B)** Iba1 positive cell density mm^2^. Same column color as graph **(A)**. Two-way ANOVA. *F*_Brain Regions_ (15, 160) = 112.7, *p* < 0.0001. *F*_Treatment_ (4, 160) = 222.1, *p* < 0.0001. *F*_Interaction_ (60, 160) = 2.039, *p* < 0.0002, and *n* = 3 mice per region. Significance: (*) Significance: respect relative vehicles. **(C)** Relative changes in percentage of area marked by Iba1. Same column color as graph **(A)**. Two-way. *F*_Brain Regions_ (15, 160) = 4.169, *p* < 0.0001. *F*_Treatment_ (4, 160) = 202.4, *p* < 0.0001. *F*_Interaction_ (60, 160) = 1.259, *p* < 0.1304, and *n* = 3 mice per region. Significance: (*) respect relative vehicles. **(D)** Relative changes in Iba1 positive cell density. Same column color as graph **(A)**. Two-way ANOVA. *F*_Brain Regions_ (15, 160) = 5.660, *p* < 0.0001. *F*_Treatment_ (4, 160) = 247.6, *p* < 0.0001. *F*_Interaction_ (60, 160) = 1.848, *p* < 0.0013, and *n* = 3 mice per region. Significance: (*) respect relative vehicles. **(E)** Representative images of Iba 1 staining in Veh, 0.05 and 5 mg/kg LPS doses in NAc, striatum, PSM cortex and cerebellum. Scale bars = 50 um in **(E)** and 10 um insets.

To examine whether microglia heterogeneity also affects the sensitivity of inflammatory responses, we performed a dose-dependent i.p. injection of LPS (from 0.005, 0.05, 0.5 to 5 mg/kg) together with the control group. Microglia morphological changes were examined 24 h after LPS administration, and we observed significant differences in several brain regions. Iba1-positive microglia marked area (Figure 3A) and cell density (Figure 3B) increased from low doses of LPS (0.05 mg/kg), particularly in the SNpr, entorhinal cortex, NAc, and hypothalamus. In most other brain regions (such as the striatum, thalamus, PSM cortex, PPA cortex, VTA, corpus callosum, and hippocampus), only 5 mg/kg LPS induced a significant increase in the marked area and the density of microglia. In the fold change analyses, we detected significant changes in the marked area at low doses in the hypothalamus, entorhinal cortex, VTA, and cerebellum, followed by SNpc, SNpr, and NAc (Figure 3C). A similar pattern was observed for the fold increase in the density of microglia cells, although the changes of this parameter were less extent and significant (Figure 3D). Similar pattern changes in cell density and marked area were observed in most other regions, which became significant at the highest dose of LPS (Figures 3C,D). This data confirms the differential sensitivity of microglia cells to LPS exposure. We next characterized the morphological features of microglia following LPS treatment (Figure 3E; Supplementary Figures 5, 6). We observed that microglia cells in the hypothalamus, SNpr, entorhinal cortex, VTA, and cerebellum exhibited activated features, e.g., enlarged cell body with thickened processes, in the mice injected with 0.05 and 0.5 mg/kg LPS dose. However, the activation did not look uniform even in the regions with activated microglia. For example, the highest microglia activation in the hypothalamus was in proximity to the CVO, while it became gradually less extensive away from this hypothalamic sub-region (Supplementary Figure 5). Then, we semi-quantitatively compared the CD68 changes in the hypothalamus, SNpr, and entorhinal cortex in mice treated with different LPS doses and the vehicle. Interestingly, we observed evident changes already in the SNpr at the dose of 0.05 mg/kg (Supplementary Figure 7). Moreover, considering relative changes, SNpr represents the only region where CD68 showed significant changes already at 0.05 mg/kg, while the hypothalamus at 0.5 mg/kg.

To confirm that the different sensitivity observed was not dependent by a different TNFα/LPS diffusion within the brain, we examined the vessel distribution using podocalyxin as a marker. We found a higher amount of vessels in the thalamus, cerebellum, and PSM, PPA, and visual cortices (Supplementary Figures 8A,B). This pattern does not correlate with the microglial sensitivity observed in several brain regions, confirming that our data were independent of TNFα and/or LPS diffusion.

This data shows that microglia cells present innate regional differences in sensitivity to systemic inflammation induced by i.p. LPS administration.

### Brain region-specific chronic inflammation 30 days after lipopolysaccharides treatment

**FIGURE 4:**
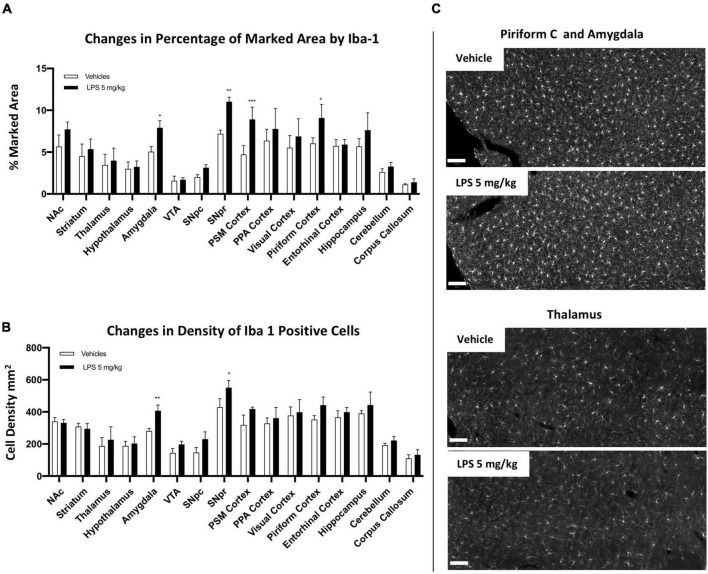
Brain regional chronic microglial activation 1 month after 5 mg/kg i.p LPS administration. **(A)** Percentage of the marked area by Iba1 in vehicles (white columns) and LPS treated mice (black columns) 30 days post administration. Two-way ANOVA. *F*_Brain Regions_ (15, 64) = 25.33, *p* < 0.0001; *F*_Treatment_ (1, 64) = 44.38, *p* < 0.0001. *F*_Interaction_ (15, 64) = 2.069, *p* < 0.0233, and *n* = 3 mice per region. Significance: (*) respect relative vehicles. **(B)** Iba1 positive cell density per mm^2^ in vehicles (white columns) and LPS treated mice 30 days post administration (black columns). Two way ANOVA. *F*_Brain Regions_ (15, 64) = 37.93, *p* < 0.0001; *F*_Treatment_ (1, 64) = 32.59, *p* < 0.0001; *F*_Interaction_ (15, 64) = 1.517, *p* < 0.1259, and *n* = 3 mice per region. Significance: (*) respect relative vehicles. **(C)** Representative images of Iba1 staining in piriform cortex, amygdala and thalamus. Scale bar = 100 um.

An interesting neuroinflammatory feature induced by systemic LPS administration is the ability to convert an acute inflammatory response to a chronic one (Qin et al., 2007; Liu et al., 2008; Zheng et al., 2013). However, whether this chronic inflammation is present uniformly in different brain regions is still not well-understood. To clarify this aspect, we treated mice with 5 mg/kg LPS and vehicle (PBS) i.p. injection and sacrificed them 30 days post-treatment. We observed a slight increase in the marked area and density of microglia cells in a few brain regions, such as the amygdala, SNpr, PSM cortex, and the piriform cortex (Figure 4; Supplementary Figures 9, 10). These data indicate sustainable microglia activation in selected brain regions in the extended period (1 month) after LPS injection. Moreover, 1 month post-LPS treatment, microglia cells lost their morphology shown 24 h after the treatment. Although a higher Iba1 intensity was still present, they adopted a more ramified morphology. This data indicates differences in microglia ability to maintain the inflammatory condition in several regions 1 month after treatment.

### Synaptic alterations due to acute and chronic neuroinflammation in SNpr

We observed that microglia cells appeared in the SNpr with the highest density in the naive mice condition and responded most severely to the acute and chronic stimulation by LPS exposure. Then, we examined the consequences on neuronal profiles in this specific region. SNpr represents an important output nucleus in basal ganglia circuitry, therefore, we examined the subsequent neuronal loss profiles in this region. SNpr consists mainly of gamma-aminobutyric acid (GABAergic) -positive neurons with a spontaneous high-frequency activity that projects to the motor thalamus (Zhou and Lee, 2011; Lanciego et al., 2012). SNpr mainly receives GABAergic projections from the striatal-nigral bundle and glutamatergic projections from the subthalamus (Lanciego et al., 2012). Moreover, this region is enriched with dopaminergic dendrites from the neighboring SNpc region, making synapses with striatal-nigral GABAergic projections (Björklund and Lindvall, 1975; Geffen et al., 1976; Mercer et al., 1979; Cheramy et al., 1981; Lanciego et al., 2012).

**FIGURE 5:**
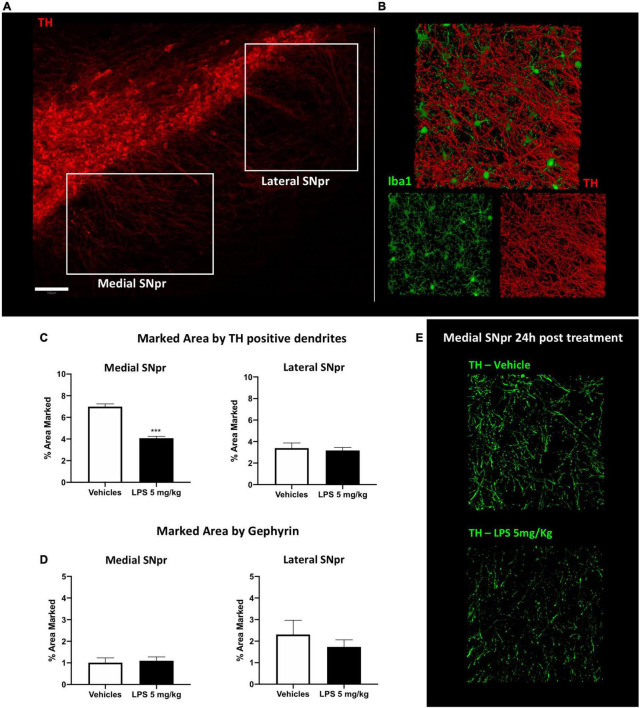
Synaptic alteration in SNpr 24 h post LPS administration. **(A)** Qualitative images of TH^+^ neurons in SNpc and their dendrites in SNpr. Scale bar = 100 um. **(B)** Qualitative confocal images of TH^+^ dendrites (red) and microglial cells in SNpr (green). **(C)** Percentage of the marked area by TH^+^ dendrites in medial and lateral SNpr 24 h after 5 mg/kg LPS or vehicle administration. Medial SNpr: Unpaired *t*-test *F*_Treatment_ 2.250, *p* < 0.0007. Lateral SNpr: Unpaired *t*-test *F*_Treatment_ 2.842, *p* < 0.7245. Significance: (***) respect relative vehicles. **(D)** Percentage of the marked area by Gephyrin in medial and lateral SNpr 24 h after 5 mg/kg LPS or vehicle administration. Medial SNpr: Unpaired t-test *F*_Treatment_ 1.469, *p* < 0.7793. Lateral SNpr: Unpaired *t*-test *F*_Treatment_ 4.027, *p* < 0.4810. **(E)** Confocal images of TH^+^ dendrites in medial SNpr 24 h after treatment vehicle and LPS treated mice.

We performed a double immune-labeling with antibodies against TH, the key enzyme for dopamine synthesis, and Iba1. We observed dopaminergic, TH-positive, dendritic profiles extending from the SNpc to the SNpr (Figure 5A), intermingled with Iba1 microglia cells branches (Figure 5B). Quantitative analyses with a series of confocal images showed that the density of dopaminergic dendrites significantly decreased in the medial part of the SNpr (Figures 5C,E) in mice exposed to 5 mg/kg LPS compared to the vehicle-treated mice 24 h after the injection. Surprisingly, this decrease was no longer evident 30 days post-LPS injection (Supplementary Figure 8C). Quantitative analyses of GABAergic synapse marker gephyrin presented no alterations in the SNpr of mice exposed to 5 mg/kg LPS (Figure 5D; Supplementary Figures 8D,E). These data suggest that the acute inflammatory response induced by LPS leads to transient and specific alterations of dopaminergic dendrites in SNpr.

### Partial ablation of CX3CR1 decreased microglia activation and prevented chronic inflammation induced by systemic lipopolysaccharides administration

**FIGURE 6:**
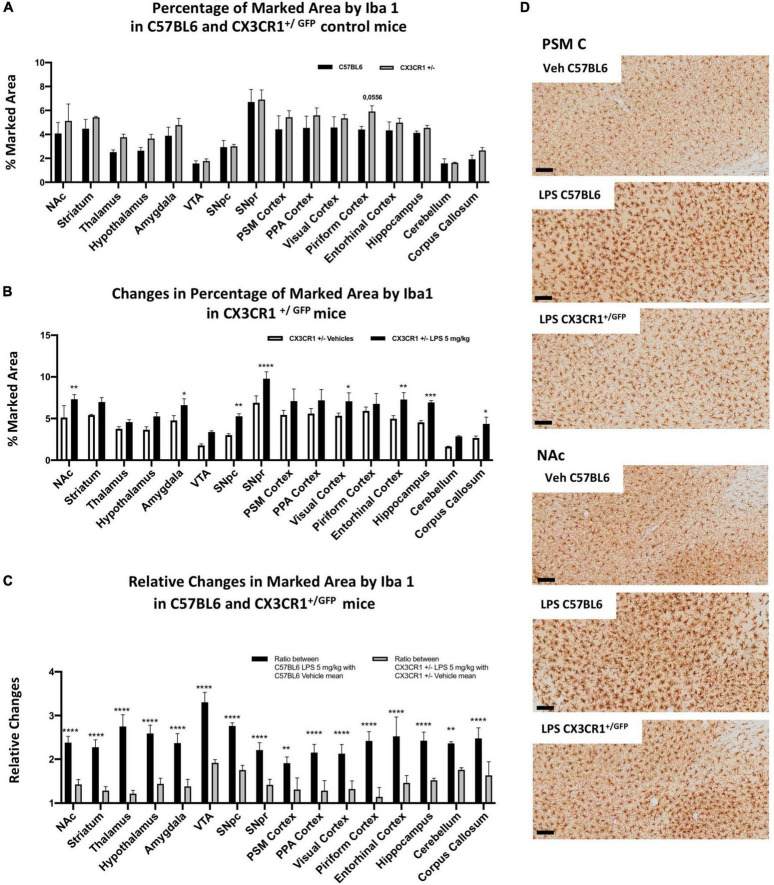
Microglia heterogeneity in CX3CR1^+/GFP^ mice. **(A)** Percentage of the marked area by Iba1 between C57BL6 (black column) and CX3CR1^+/GFP^ mice (gray columns) in control conditions. Two-way ANOVA. *F*_Brain Regions_ (15, 64) = 32.50, *p* < 0.0001. *F*_Genotype_ (1, 64) = 36.68, *p* < 0.0001. *F*_Interaction_ (15, 64) = 0.7510, *p* < 0.7241, and *n* = 3 mice per region. Significance: (*) respect same regions between two genotypes. **(B)** Percentage of the marked area by Iba1 positive cells in CX3CR1^+/GFP^ mice. Vehicles (white columns) and LPS treated mice (black columns). Two-way ANOVA. *F*_Brain Regions_ (15, 64) = 34.78, *p* < 0.0001. *F*_Treatment_ (1, 64) = 164.9, *p* < 0.0001. *F*_Interaction_ (15, 64) = 1.327, *p* < 0.2129, and *n* = 3 mice per region. Significance: (*) respect relative vehicles. **(C)** Relative changes in percentage of the marked area by Iba1 between LPS 5 mg/kg and vehicles mice 24 h after treatment in C57BL6 mice (black columns) and CX3CR1^+/GFP^ mice (gray columns). Two-way ANOVA *F*_Brain Regions_ (15, 64) = 10.13, *p* < 0.0001. *F*_Treatment_ (1, 64) = 687.5, *p* < 0.0001. *F*_Interaction_ (15, 64) = 2.862, *p* < 0.0018, and *n* = 3 mice per region. Significance: (*) respect relative vehicles. **(D)** Representative images of Iba1 staining in PSM cortex and NAc in C57BL6 and CX3CR1^+/GFP^ mice treated with 5 mg/kg LPS or vehicles. Scale bar = 100 um.

CX3CR1 is a chemokine receptor localized in microglia and other immune cells, such as dendritic cells. Its ligand, CX3CL1 (fractalkine), is constitutively expressed in neurons (Pawelec et al., 2020). CX3CR1^+/GFP^ mice have been widely used in studying microglia dynamics and activation in various models of neurodegenerative diseases (Marker et al., 2010; Avignone et al., 2015). We, therefore, investigated microglia alterations in CX3CR1 partially ablated mice (CX3CR1^+/GFP^). We first examined C57BL6 and CX3CR1^+/GFP^ age-matched mice in the naive condition (without LPS exposure). Comparing the percentage of the marked area by Iba1, we found no significant differences between wild type and CX3CR1^+/GFP^ mice (Figure 6A), indicating that microglia seem to be similar between these two mice in the resting condition, at least from a morphological point of view. Subsequently, we analyzed CX3CR1^+/GFP^ mice treated with 5 mg/kg LPS and vehicle 24 h post-injection (Figure 6B). Significant microglia activation was present in SNpr, SNpc, NAc, amygdala, visual cortex, entorhinal cortex, and hippocampus, while not appearing in the rest of the brain regions. Comparing relative changes between LPS- and vehicle-treated groups in C57BL6 and CX3CR1^+/GFP^ mice, we observed a general and significant decrease in relative changes in all regions examined in mice partially ablated by CX3CR1 (Figures 6C,D; Supplementary Figures 11, 12). These data suggest that partial ablation of this receptor made the microglia cell less sensitive or responsive to the LPS treatment.

To further confirm this observation, we qualitatively compared CD68 changes. No differences were observed between C57BL6 and CX3CR1^+/GFP^ mice in the control condition. Comparing CX3CR1^+/GFP^ mice treated with 5 mg/kg of LPS and relative controls, we observed a slight or no increase in CD68 signal. Comparing CD68 between C57BL6 and CX3CR1^+/GFP^ mice treated with 5 mg/kg LPS, we observed a clear lower CD68 expression in CX3CR1^+/GFP^ mice (Supplementary Figure 13).

**FIGURE 7:**
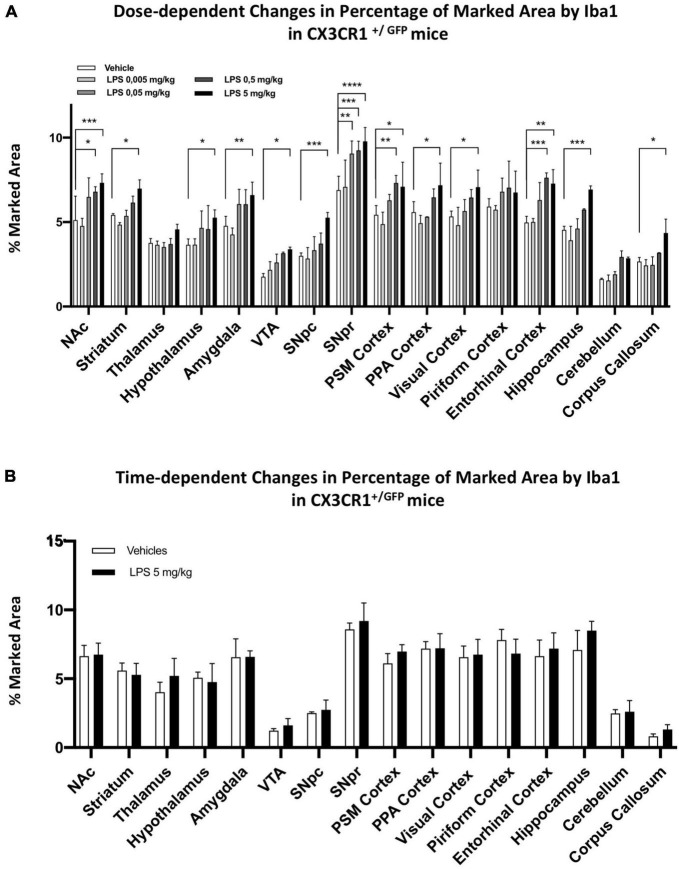
Dose and time-dependent microglia response in CX3CR1^+/GFP^ mice. **(A)** Percentage of marked area by Iba1 in vehicles (white columns), LPS treated mice with 0.005 mg/kg (clear gray columns), 0.05 mg/kg (medium gray columns), 0.5 mg/kg (dark gray columns), 5 mg/kg (black columns) 24 h after treatment. Two-way ANOVA. *F*_Brain Regions_ (15, 160) = 97.53, *p* < 0.0001. *F*_Treatment_ (4, 160) = 77.01, *p* < 0.0001. *F*_Interaction_ (60, 160) = 1.219, *p* < 0,1668, and *n* = 3 mice per region. Significance: (*) respect relative vehicles. **(B)** Percentage of marked area by Iba1 in vehicles (white columns) and 5 mg/kg LPS (black columns) 30 days after treatment. Two-way ANOVA. *F*_Brain Regions_ (15, 160) = 50.73, *p* < 0.0001. *F*_Treatment_ (1, 64) = 4.453, *p* < 0.0388. *F*_Interaction_ (15, 64) = 0.7369, *p* < 0.7385, and *n* = 3 mice per region. Significance: (*) respect relative vehicles.

We then examined the effect of partial ablation of CX3CR1 on the dose-dependent pattern observed in C57BL6 mice (Figure 7A). We treated C57BL6 and CX3CR1^+/GFP^ mice with 5, 0.5, 0.05, and 0.005 mg/kg i.p. LPS and relative vehicle. We observed that the partial ablation of CX3CR1 led to a general decrease in microglia activation upon systemic LPS administration. Despite a general decreased response at lower doses, microglia in CX3CR1^+/GFP^ mice were responsive to the high dose (5 mg/kg) LPS treatment in most brain regions tested, except for the thalamus, piriform cortex, and cerebellum. SNpr represented the brain region with the highest microglia sensitivity, which became significant at 0.05 mg/kg. A recent study compared the effects of LPS in SNpr of C57BL6 mice between the doses of 1 and 5 mg/kg i.p. injections. It appeared that only the higher dose could sustain the chronic microglia activation (Zhao et al., 2020). Here, we examined whether microglia activation observed in CX3CR1^+/GFP^ 24 h after the LPS treatment can be sustained for the long term. Thirty days after LPS injection, microglia activation was not observed in CX3CR1 partially ablated mice. No differences could be observed in all the brain regions tested between LPS-treated and vehicle-treated mice (Figure 7B). This data implied that CX3CR1 partial ablation decreased sensitivity to microglia activation and sustainability in response to systemic LPS administration.

### Brain region-specific astrocytic activation in response to systemic lipopolysaccharides administration

We have also investigated astrocytic heterogeneity, which represents another essential cell type in neuroinflammation. These cells cooperate with microglial cells in the neuroinflammatory event. Astrocytes could also have a pro-inflammatory toxic phenotype and harm the surrounding tissue (Miller, 2018; Linnerbauer et al., 2020).

**FIGURE 8:**
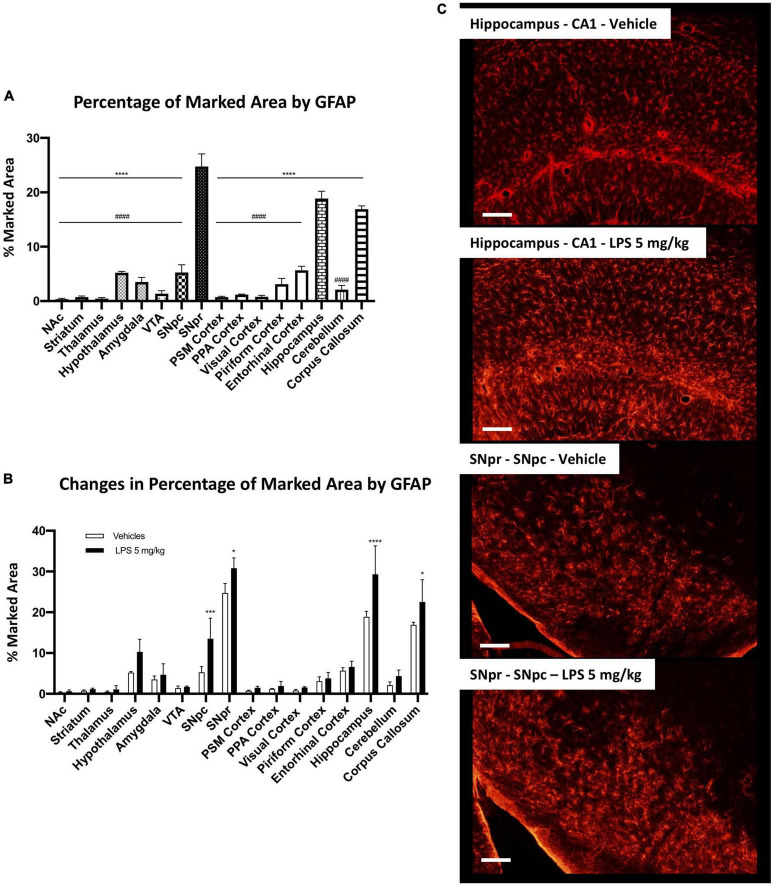
Brain region-specific astrocyte distribution and activation induced by systemic LPS. **(A)** Percentage of the marked area by GFAP positive astrocytes in vehicles C57BL6N mice. One-way ANOVA *F*_Brain Regions_ (15, 32) = 206.8, *p* < 0.0001, and *n* = 3 mice per region. Significance: (*) respect SNpr; (#) respect hippocampus and corpus callosum. Other significances are summarized in Supplementary Table 5. **(B)** Percentage of the marked area by GFAP positive cells in vehicles (white columns) and LPS treated mice (black columns). Two-way ANOVA *F*_Brain Regions_ (15, 64) = 100.6, *p* < 0.0001. *F*_Treatment_ (1, 64) = 39.24, *p* < 0.0001. *F*_Interaction_ (15, 64) = 3.406, *p* < 0.0003, and *n* = 3 mice per region. Significance: (*) respect relative vehicles. **(C)** Representative images of GFAP positive astrocytes in hippocampus and SNpr in vehicles and LPS treated mice.

Firstly, we examined the distribution of astrocytes using the glial fibrillary acid protein (GFAP) as a marker. Although these cells are distributed in all brain parenchyma, they express different levels of GFAP among the different regions. For this reason, it is not possible to equally mark these cells within all regions, and their heterogeneity could be highly influenced by the different expression of this marker between the different astrocytic populations. We analyzed the marked area by GFAP positive signal in C57BL6 mice in the vehicle condition. SNpr, hippocampus, and corpus callosum presented a significantly higher density of GFAP positive cells (Figure 8A). Moreover, the morphology of these cells appeared different among different brain regions.

Then, we compared C57BL6 mice treated with 5 mg/kg LPS and the vehicle control. This analysis showed a heterogeneous increase in GFAP signal between several brain regions. We observed a significant astrocyte increase in the hippocampus, SNpc, SNpr (Figures 8B,C), and corpus callosum (Supplementary Figure 14A), but not in the other areas. Further alterations were qualitatively observed. GFAP signal increases in the hypothalamus, but intraregional differences mitigate this increase. An increase in GFAP signal was also observed in other regions such as the cerebellum (Supplementary Figure 14B) and the thalamus. However, the GFAP intensity was below the threshold used in the analyses, and the increase was not detected.

**FIGURE 9:**
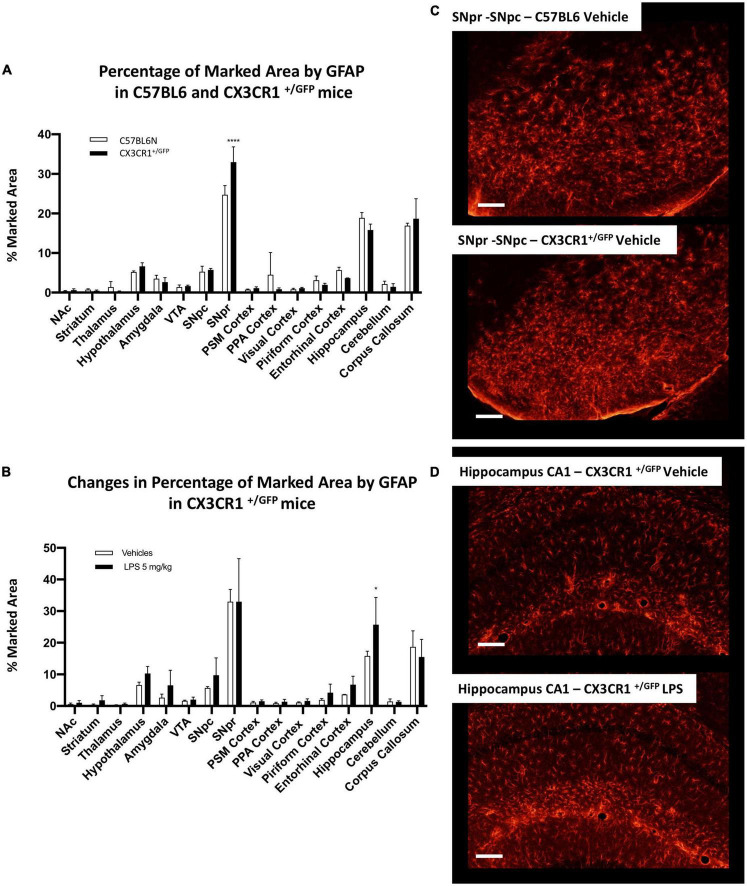
Astrocytes heterogeneity in CX3CR1^+/GFP^ mice. **(A)** Percentage of marked area by GFAP positive astrocytes in vehicles C57BL6N and CX3CR1^+/GFP^ mice. Two-way ANOVA *F*_Brain Regions_ (15, 64) = 136.9, *p* < 0.0001. *F*_Genotype_ (1, 64) = 0.00128, *p* < 0.9715. *F*_Interaction_ (15, 64) = 3.575, *p* < 0.0002, and *n* = 3 mice per region. Significance: (*) respect relative regions in C57BL6 mice. **(B)** Changes in percentage of marked area by GFAP positive astrocytes in CX3CR1^+/GFP^ mice cells in vehicles (white columns) and LPS treated (black columns). Two-way ANOVA. *F*_Brain Regions_ (15, 64) = 39.36, *p* < 0.0001. *F*_Treatment_ (1, 64) = 5.526, *p* < 0.0218. *F*_Interaction_ (15, 64) = 0.9605, *p* < 0.5055, and *n* = 3 mice per region. Significance: (*) respect relative vehicles. **(C)** Confocal images of GFAP positive astrocytes in SNpr in C57BL6 and CX3CR1^+/GFP^ mice. **(D)** Representative images of GFAP positive astrocytes in hippocampus of CX3CR1^+/GFP^ mice treated with 5 mg/kg LPS and relative control.

Comparing GFAP-positive cells between C57BL6 and CX3CR1^+/GFP^ mice, we observed a similar GFAP pattern in the naive (non-LPS treated) mice (Figure 9A). The only exception was a significant increase in GFAP marked area in SNpr in CX3CR1^+/GFP^ mice. Further qualitative observations revealed that this increase appeared to involve mainly in the medial part of SNpr and it seems to be due to a higher astrocytes density (Figure 9C). In the end, we investigated astrocytic activation in CX3CR1^+/GFP^ mice treated with 5 mg/kg of LPS compared to the vehicle treated mice. This analysis detected an increase in GFAP signal only in the hippocampus (Figures 9B,D) but not in SNpr (Supplementary Figure 14C).

Our data reveal that astrocytes also present a brain region-specific activation and confirmed a lower inflammatory response in CXRCR1 partially ablated mice compared to the mice that express a physiological amount of this receptor.

## Discussion

Microglia cells represent the immune resident cell population of the CNS, which constantly survey the brain parenchyma and actively participate in tissue homeostasis (Nimmerjahn et al., 2005; Paolicelli et al., 2011; Parkhurst et al., 2013). Several studies have shown that variations in morphology, density, and transcriptomes of microglia cells are dependent on their location in the brain (Lawson et al., 1990; de Haas et al., 2008; Grabert et al., 2016; De Biase et al., 2017; Ayata et al., 2018; Abellanas et al., 2019; Masuda et al., 2020). In the present study, we aimed: (1) to understand whether microglia heterogeneity could lead to differential inflammatory responses within the brain; (2) to explore astrocyte heterogeneity; (3) to examine the impact of inflammation in SN; (4) to investigate the effect of CX3CR1 partial ablation in a model of systemic inflammation. Our study reports an extensive analysis of microglial and astrocytes cells and their inflammatory activation in 16 brain regions: eight nuclei, seven different cortices, and one area of white matter. Using a holistic approach, we have revealed brain regions with peculiar inflammatory characteristics that could be important for the pathological progression of several neurodegenerative diseases.

### Brain region-specific microglia inflammation

We first investigated microglia heterogeneity in terms of the marked area, cell density, and morphology in control conditions between the 16 different brain regions analyzed. Our data showed that nine areas were similar, while the other seven appeared different.

To explore microglia activation under systemic inflammation, we used a mouse model treated with a single i.p. injection of 5 mg/kg LPS. This model showed differences in microglia activation in several brain regions and the strongest increase of Iba1 expression was observed in SNpr and entorhinal cortex. Further qualitative analyses about the alterations on the lysosomal system in several microglial populations showed the most evident changes in SNpr.

However, investigating Iba1 relative changes we observed that SNpr is less subject to an increase of microglia density compared to the entorhinal cortex and other brain regions. This finding agrees with the data obtained by Abellanas and colleagues. They showed that the midbrain is the only area compared with the striatum, cortex, and hippocampus where it is possible to detect an increase of anti-inflammatory cytokines transforming growth factor-beta (TGFβ) and IL10 expression 48 h after LPS administration (Lynch et al., 2004; Arimoto et al., 2007; Tesseur et al., 2017; Abellanas et al., 2019). Abellanas, in his work, suggested the presence of negative feedback that can down-regulate the neuroinflammatory response in this area. This feedback could have a crucial role in containing the inflammatory response under a certain limit to preserve the surrounding tissue. Comparing 5 mg/kg LPS and vehicles groups, we found higher microglia relative changes in regions characterized by low microglia density in control conditions. Whether these different responses represent compensatory mechanisms it is not clear, but it confirms the presence of heterogeneous inflammatory responses between different brain regions.

Using a dose-dependent paradigm, we detected different levels of microglia sensitivity to systemic TNFα/LPS. The mechanisms behind the different microglia sensitivity observed are unknown, and further studies are needed to investigate these aspects. Moreover, these mechanisms may differ between several regions. According to our data regarding the relative changes in Iba1, the most sensitive microglia population was in the hypothalamus. In this region, in proximity to the CVOs, we observed activated microglia cells at the lowest LPS concentrations. Here, the lack of BBB exposes this cell population to the systemic bloodstream and pro-inflammatory agents more effectively than in other brain regions. The effects observed in this region could derive from higher exposure to TNFα and LPS and the synergic activations of their receptors (Vargas-Caraveo et al., 2017, 2020; Furube et al., 2018). Interestingly, different works described that microglia cells in this region could modulate hypothalamic functions, such as regulating the body metabolism (Rosin and Kurrasch, 2019; Valdearcos et al., 2019).

Our data on the dose-dependent microglia response indicates that also the microglia population in the SNpr is particularly sensitive compared to most of other regions. Abellanas’s study showed that a higher portion of microglia cells in the SNpr express pro-inflammatory receptors such as TLR4 with respect to the other regions analyzed. Furthermore, De Biase summarized their findings arguing that microglia cells in SNpr have an “injury-responsive phenotype” (De Biase et al., 2017; Abellanas et al., 2019).

Our results indicate microglial ability to perceive and react to a systemic pro-inflammatory stimulus. More studies are needed to understand whether the pattern observed could also reflect microglial cells’ sensitivity when pro-inflammatory agents are within the brain parenchyma. An example could be represented by microglial sensitivity observed in the entorhinal cortex. Our findings agree with previous observations in Alzheimer’s disease (AD) models, where microglia activation in this cortex precedes all other regions as well as amyloid plaques deposition (Janelsins et al., 2005).

Interestingly, we observed evident CD68 changes at 0.05 mg/kg LPS only in SNpr compared with the hypothalamus and entorhinal cortex, which showed an increase at the higher doses. This observation confirmed different sensitivity in microglial activation among different brain regions. However, it also pointed out that several patterns could be found using different markers. These differences could be due to different microglia pro-inflammatory phenotypes and a specific protein’s role in a particular brain region. Recently, Zhao showed that chronic inflammation depends on IL1β expression, which is prevented in the nucleotide-binding domain and leucine-rich repeat receptor containing a pyrin domain 3 (NLRP3) and IL1β deficient mice (Zhao et al., 2020). Other authors showed different IL1β expression patterns under systemic inflammatory conditions in several brain areas (Silverman et al., 2014; Abellanas et al., 2019), suggesting that also chronic inflammation could differ throughout the brain. Our data shows that this is indeed the case. We compared 5 mg/kg LPS and relative control mice 30 days after treatments, and we found a slight increase in the microglia marked area only in some regions such as the amygdala, SNpr, PSM cortex, and piriform cortex. However, microglia cells partially lost the pro-inflammatory morphology shown 24 h after LPS injection, assuming an intermediate state. Altogether, our data showed that microglia heterogeneity leads to different inflammatory responses when systematically stimulated with TNFα/LPS. Recently, systemic inflammation has been under the spotlight concerning the COVID-19 pandemic. High levels of TNFα in the bloodstream and the development of chronic neurological symptoms were widely described in the past year (Del Valle et al., 2020; Feldmann et al., 2020; Robinson et al., 2020; Marshall, 2021). Interestingly, no trace of COVID-19 was found within the brain (Marshall, 2021). Whether these symptoms are related to the systemic “storm” of cytokines during the acute stage of COVID-19 infection and the subsequent neuroinflammatory activation is still not understood.

Most of the regions identified in our work with distinct microglia responses are also involved in the two most common neurodegenerative diseases. The entorhinal cortex, piriform cortex, amygdala, and NAc are all regions involved in AD pathology, while the SN is the region mainly affected in Parkinson’s disease (PD) and related disorders. Our data suggest that the different neuroinflammatory responses could partially influence the regional neurodegenerative pattern observed in different neurodegenerative diseases.

### The neuroinflammatory process in *SNpr* leads to synaptic changes affecting the striato-nigral pathway

Neurodegeneration observed within the SNpc of PD patients and other related disorders are mainly associated with losing dopaminergic neurons. This neuronal population is more sensitive to reactive oxygen species (ROS), which exacerbate the detrimental effect of pathological agents such as α-synuclein or toxins (Drechsel and Patel, 2008; Dutta et al., 2008; Gao et al., 2008; Ramsey and Tansey, 2013; Rai and Singh, 2020; Rai et al., 2020). Moreover, it has been shown that ROS species, LPS, and pro-inflammatory cytokines such as TNFα and IL1β, can trigger the activation of several transcription factors, such as nuclear factor- kb and transcription factor EB (Brady et al., 2018; Rai et al., 2021). These transcription factors in turn regulate the expression of inflammatory cytokines and autophagy system, which is crucial for several neurodegenerative diseases, such as PD and AD (Cortes and La Spada, 2019; Rai et al., 2021). In the present study, we showed that the neuroinflammatory response in SNpr can induce a rapid alteration of dopaminergic dendrites in the medial SNpr 24 h post-LPS injection. These data indicate that the inflammatory process in SNpr could directly affect the neuronal population in the neighboring SNpc region. Moreover, our data showed that this decrease in dendrites is reversible and no longer visible 1 month after LPS administration. This finding is in line with observations made by Ong et al. (2021) regarding the increase in TH activity 1 week after peripheral LPS administration. The authors did not identify an apparent reason for this early increase in TH activity. Here, we showed that it could be related to the re-establishment of the dopaminergic dendritic profile in SNpr after the damages induced by the acute inflammatory response. SNpr consists of neurons less densely compacted compared to those in the SNpc and VTA, and the region is particularly enriched by neurites from other regions and glial cells. We observed that dopaminergic dendrites in this region surround microglia cells. An interesting characteristic described is that these dendrites release dopamine in the SNpr (Björklund and Lindvall, 1975; Geffen et al., 1976; Mercer et al., 1979; Cheramy et al., 1981), which was suggested to possesses a modulatory effect on the GABAergic neurons (Waszcak and Walters, 1983; Martin and Waszczak, 1996; Windels and Kiyatkin, 2006). However, other works described the ability of this neurotransmitter to modulate also microglia cell activity (Farber et al., 2005; Yan et al., 2015; Elgueta et al., 2017; Fan et al., 2018). In this region, the inflammatory response presented the most robust activation, one of the highest sensitivities to systemic inflammation, and the ability to maintain chronic microglial activation. Additionally, other evidence points to the fact that this cell population seems to be regulated by several feedback mechanisms. These characteristics highlight the complexity and the peculiarity of microglia cells in SNpr and bring us to consider a possible physiological role. Many studies showed that microglia cells are essential for synapse modeling within the brain (Kim et al., 2013; Ikegami et al., 2019; Crapser et al., 2021). Here, we observed a rapid and transient alteration of dopaminergic dendrites with further consequences on the striato-nigral bundle. We speculate that SNpr could represent the “bridge” between the immune system and basal ganglia circuitry, which may have a physiological role in modulating its structure and activity.

### CX3C axis as a pharmacological target for neuroinflammation

During the last two decades, an increased number of studies focused on the functions of the fractalkine axis CX3CL1-CX3CR1. CX3CL1 is constitutive and abundantly expressed in neurons, while its receptor is exclusively expressed in microglia cells in the CNS and other peripheral immune cells, such as dendritic cells (Pawelec et al., 2020). Two-photon *in vivo* imaging demonstrated microglial dynamics and its interaction with neuronal profiles, such as dendrites, axons, and synapses in mice expressing both CX3CR1^+/GFP^ and Thy1-YPF. Moreover, mice completely ablated for CX3CR1 presented aberrant synaptic connections between neurons, indicating the crucial role of microglia cells in synaptic modulation (Paolicelli et al., 2011).

Many studies also showed the role of the CX3C axis in physiology and in several neurodegenerative diseases (Biber et al., 2007; Lyons et al., 2009; Wolf et al., 2013). Unlike other chemokines, CX3C is more highly expressed in the CNS than in the periphery and, for this reason, it could represent a crucial pharmacological target for neurodegenerative diseases. However, contradictory data were obtained when this axis was perturbed in models of different neurodegenerative diseases (Pawelec et al., 2020). In models of stroke, epilepsy, and amyotrophic lateral sclerosis, a decrease of CX3CR1 or a rise of its ligand seem to be protective (Soriano et al., 2002; Cipriani et al., 2011; Yeo et al., 2011; Xu et al., 2012; Fumagalli et al., 2013; Tang et al., 2014; Liu et al., 2019). The deficiency of CX3CL1 or CX3CR1 in AD models reduces β-amyloid deposition but increases tau phosphorylation and aggregation, with a consequent detrimental effect on the behavioral and cognitive deficit (Bhaskar et al., 2010; Fuhrmann et al., 2010; Lee et al., 2010, 2014; Liu et al., 2010; Maphis et al., 2015; Merino et al., 2016). In PD models based on α-synuclein over-expression, the deficiency of this receptor has led to contradictory results. The increase of CX3CL1 induced protection to dopaminergic neurodegeneration (Pabon et al., 2011; Morganti et al., 2012; Nash et al., 2015; Thome et al., 2015; Castro-Sánchez et al., 2018). Pabon et al. (2011) showed that the administration of exogenous CX3CL1 in rats treated with 6-hydroxydopamine (6-OHDA) was protective. An absence of dopaminergic neurodegeneration was observed in CX3CR1 deficient mice treated with intranasal inoculation of 6-OHDA or 1-methyl-4-phenyl-1,2,3,6-tetrahydropyridine (MPTP). On the contrary, extensive neurodegeneration was observed in CX3CR1 deficient mice treated with an i.p. injection of MPTP. They found a higher activation of astrocytes than that in control mice (Tristão et al., 2016).

Comparing mice partially and totally ablated of CX3CR1, Cardona et al. (2006) found that the complete ablation increased neuroinflammatory effects and neurodegeneration mediated by systemic LPS. However, they did not compare these models with mice expressing physiological levels of CX3CR1, and it is unclear whether the differences observed are due to a real increase in microglial response in mice totally ablated or by a decrease in mice that partially express this receptor.

Here, we showed that the partial ablation of CX3CR1 can significantly decrease microglia response when mice are treated with 5 mg/kg LPS and compared with mice that express physiological levels of CX3CR1. Moreover, chronic microglial activation was not sustained in all regions analyzed 30 days after LPS administration. These differences between partial and total ablation may be due to the partial decrease of CX3CR1 without altering the amount of CX3CL1, leading to changes in the ligand-receptor ratio, with consequences in their binding equilibrium. These data suggest the importance of CX3C axis integrity and that a higher amount of CX3CL1 respect CX3CR1 could depress the inflammatory response. Nevertheless, mice partially ablated of CX3CR1 can still respond to LPS stimulus in the acute experimental paradigm, which indicates the usefulness of such a model in microglia optical imaging. However, one should also be aware of the potential insensitivity to pro-inflammatory stimuli. These data also imply the CX3C axis as a potential pharmaceutical target in neurological and neurodegenerative diseases.

### Brain region-specific astrocytic activation

Astrocytes are another cell type that cooperates with microglia during a neuroinflammatory event. Several studies showed their involvement in several animal models, including those investigated in our study (Miller, 2018; Linnerbauer et al., 2020). To have a complete overview of the inflammatory heterogeneity within the brain, we also briefly investigated this cell type. Astrocytes appeared highly heterogeneous regarding GFAP expression, cell density, and morphological characteristics. SNpr, hippocampus, and corpus callosum represent the regions most densely populated by astrocytes. Moreover, astrocytes in these regions increase significantly under inflammatory conditions induced by 5 mg/kg of LPS.

Comparing C57BL6 with CX3CR1 partial ablated mice, we observed a significant higher GFAP positive signal in SNpr, which seems to be due to an increase in astrocytes density in the medial part of this region. Moreover, we found significant astrocytic increase only in the hippocampus by investigating the inflammatory changes induced by 5 mg/kg of LPS in CX3CR1^+ ⁣/^*^GFP^* mice and relative control. However, this increase was significantly lower than the one observed in C57BL6, confirming that partial ablation of the CX3CR axis also decreases astrocytic activation.

## Conclusion

This work investigated whether microglia and astrocytes heterogeneity leads to different inflammatory responses. Our data showed that this is indeed the case for a pro-inflammatory stimulus, such as LPS. Our results underline the peculiar microglial activation in SNpr and its ability to affect the dopaminergic dendrites, altering selectively the striato-nigral bundles. In the end, we identified the CX3C axis as a possible pharmacological target for neuroinflammation induced by an increase of systemic TNFα and/or LPS. Nevertheless, we are aware of the limitation of the present study. With references of previous studies using techniques of RNAseq, transcriptomics, and proteomics, our study complementarily provides detailed evidence on morphological heterogeneity of microglial and astrocytes distribution and activation among different brain regions in response to acute and chronic exposure of LPS.

## Data availability statement

The original contributions presented in this study are included in the article/Supplementary material, further inquiries can be directed to the corresponding author.

## Ethics statement

The animal study was reviewed and approved by Malmö/Lund Animal Ethical Committee (Dnr 5.8.18-09454/2021).

## Author contributions

EB contributed to the conception and design of the study and performed the animal experiments and statistical analysis. J-YL supervised the design of the study. EB and LT-G prepared the samples. EB, CH, and LT-G performed the staining. EB, AS, and LT-G performed images acquisition and image analysis. EB and J-YL wrote the manuscript. All authors contributed to the project and manuscript revision and approved the submitted version.

## Supplementary material

Supplementary Figure 1 **(A)** Qualitative examples about ROI sampling in some sections. The legend for colors is located under the respective images. The scanned sections are labeled with Iba1. **(B)** Qualitative images about the microglial marked area analyses. The image show outlines around soma and main branches. Section stained with Iba1. **(C)** Qualitative images about the microglial density analyses. The image show outlines exclusively around the soma. Section stained with Iba1.

Supplementary Figure 2 3D confocal images of microglial cells in all 16 regions examined. Sections labeled with Iba1-Cy3 and acquired with 63x magnification with z-stack of 11 um with 0.5 um increment between optical sections. Scale bar = 50 um.

Supplementary Figure 3 Qualitative images of CD68 staining in different brain regions in control C57BL6 mice. This panel shows the different CD68 distribution in NAc, hypothalamus, SNpr, VTA, PSM cortex, entorhinal cortex, hippocampus, and cerebellum. It is possible to appreciate the higher amount of vesicle in SNpr in respect to all other regions. Scale bars = 20 um.

Supplementary Figure 4 Alterations of CD68 positive cells in response to the treatment of LPS. **(A)** Change in percentage marked area by CD68 immunoreactivity among different brain regions. Two-way ANOVA. *F*_Brain Regions_ (7, 32) = 32.12, *p* < 0.0001. *F*_Treatment_ (1, 32) = 107.3, *p* < 0.0001. *F*_Interaction_ (7, 32) = 2.631, *p* = 0.0289, and *n* = 3 mice per region. Significance: (*) respect relative vehicles. **(B)** The relative changes in the marked areas between the vehicle- and LPS 5mg/kg treatments among different brain regions. One way ANOVA *F*_Brain Regions_ (7, 16) = 8.982, *p* = 0.0002, and *n* = 3 mice per region. Significance: (*) respect SNpc; (#) respect VTA. **(C)** Representative images of CD68 staining in different brain regions (SNpr, VTA, and PSM cortex) in vehicle- and LPS (5 mg/kg) treated C57BL6 mice. It is evident that CD68 immunoreactivity is higher in SNpr compared to other brain regions. Scale bars = 20 um.

Supplementary Figure 5 Qualitative images of dose-dependent microglial activation in mice treated with 0.05 and 5 mg/kg of LPS and relative vehicles. This panel of images shows the different microglial sensitivity and activation in the hypothalamus compared with all other regions in the same section. Moreover, panels **(D,E)** represent good examples of intra-regional differences. Microglia activations within the hippocampus seem to be more sensitive in CA1 and subiculum respect to CA3 and gyrus dentatus. **(A)** Scale bar = 800 um. **(B,C)** High power images of the thalamus and hypothalamus. **(B)** Scale bar = 100 um; **(C)** scale bar = 20 um. **(D,E)** High power images of the hippocampus in the same section. **(D)** Scale bar = 100 um; **(E)** high power images of the CA1 and CA3. Scale bar = 20 um.

Supplementary Figure 6 Qualitative images of dose-dependent microglial activation in mice treated with 0.05 and 5 mg/kg of LPS and relative vehicles. This panel of images shows the different microglial sensitivity between SNpr, VTA, and anterior entorhinal cortex with respect to visual cortex. **(A)** Scale bar = 500 um. **(B,C)** High power images of SNpr and VTA in the same section. **(B)** Scale bar = 200 um; **(C)** scale = bar 20 um. **(D,E)** High power images of visual and entorhinal cortex in the same section. **(D)** Scale bar = 100 um. **(E)** Scale bar = 20 um.

Supplementary Figure 7 Alterations of CD68 in response to different doses of LPS treatments. **(A)** Change in percentage marked area by CD68 immunoreactivity in vehicles (white columns), LPS treated mice with 0.05 mg/kg (medium gray columns), 0.5 mg/kg (dark gray columns), 5 mg/kg (black columns). Two-way ANOVA. *F*_Brain Regions_ (2, 24) = 79.43, *p* < 0.0001. *F*_Treatment_ (3, 24) = 13.26, *p* < 0.0001. *F*_Interaction_ (6, 24) = 1.787, *p* < 0.1443, and *n* = 3 mice per region. Significance: respect relative vehicles. **(B)** The relative changes in the marked areas between the vehicle- and different doses of LPS (0.05-5mg/kg) treatments among different brain regions. The same column color as graph (A). Two-way ANOVA with Tukey correction. *F*_Brain Regions_ (2, 24) = 4.131, *p* < 0.0001. *F*_Treatment_ (3, 24) = 14.11, *p* < 0.0287. *F*_Interaction_ (6, 24) = 0.8165, *p* < 0,5678, and *n* = 3 mice per region. Significance: (*) respect relative vehicles. **(C)** Representative images of CD68 staining in the dose-dependent in mice treated with 0.05 mg/kg and 5 mg/kg of LPS and relative vehicles. This panel shows the increase of CD68 in hypothalamus, SNpr and entorhinal cortex. CD68 immunoreactivity appears more robust in the low dose LPS exposure in SNpr compared to other brain regions. Scale bars = 20 um.

Supplementary Figure 8 **(A)** Percentage of the marked area by podocalyxin in vehicles C57BL6N mice. One-way ANOVA *F*_Brain Regions_ (15, 32) = 11.69, *p* < 0.0001, and *n* = 3 mice per region. Significance: (*) respect the cerebellum; (#) respect the thalamus. Other significances are summarized in Supplementary Table 6. **(B)** Qualitative images of podocalyxin staining in thalamus, SNpr, and the entorhinal cortex in vehicle C57BL6 mice. **(C)** Percentage of marked area by TH-positive dendrites in medial and lateral SNpr 30 days post LPS injection. Medial SNpr: Unpaired t-test *F*_Treatment_ 4.796, *p* < 0.3450. Lateral SNpr: Unpaired t-test *F*_Treatment_ 2.171, *p* < 0.6308. **(D)** Percentage of marked area by Gephyrin-positive nerve terminals in medial and lateral SNpr 30 days post LPS injection. Medial SNpr: Unpaired t-test *F*_Treatment_ 4.743, *p* < 0.3483. Lateral SNpr: Unpaired t-test *F*_Treatment_ 4.349, *p* < 0.3739. **(E)** Representative images of Gephyrin-positive profiles in medial SNpr in Vehicle and LPS treated mice 30 days post-injection.

Supplementary Figure 9 Qualitative images of chronic inflammation in mice treated with 5 mg/kg and relative vehicles. This panel of images shows the different microglia activation 30 days after LPS administration in the PSM cortex with respect to all other regions in the same section. **(A)** Scale bars = 500 um. **(B,C)** High power images of PSM cortex, corpus callosum, striatum, and NAc in the same sections. **(B)** Scale bars = 100 um. **(C)** Scale bars = 20 um.

Supplementary Figure 10 Qualitative images of chronic inflammation in mice treated with 5 mg/kg and relative vehicles. This panel of images shows a different microglia activation 30 days after LPS administration in SNpr and hippocampus with respect to the VTA and visual cortex in the same section. **(A)** Scale bars = 500 um. **(B)** High power images of SNpr and VTA in the same sections. Scale bars = 200 um. **(C)** High power images of the hippocampus and visual cortex in the same sections. Scale bars = 100 um. **(D)** High power images of SNpr, VTA, CA1, and visual cortex in the same sections. Scale bar 20 um.

Supplementary Figure 11 Qualitative images of inflammation in C57BL6 and CX3CR1^+/GFP^ mice treated with 5 mg/kg and relative vehicles for 24 h. This panel of images shows the lower microglia activation in CX3CR1^+/GFP^ mice with respect to C57BL6 mice. **(A)** Scale bars = 800 um. **(B,C)** High power images of the thalamus and hypothalamus in the same sections. **(B)** Scale bars = 100 um. **(C)** Scale bars = 20 um. **(D,E)** High power images of hippocampus in the same sections. Scale bars = 100 um. **(E)** High power images CA1 and CA3 in the same sections. Scale bars = 20 um.

Supplementary Figure 12 Qualitative images of inflammation in C57BL6 and CX3CR1^+/GFP^ mice treated with 5 mg/kg and relative vehicles for 24 h. This panel of images shows the lower microglia activation in CX3CR1-GFP mice with respect to C57BL6 mice. **(A)** Scale bars = 500 um. **(B,C)** High power images of SNpr and VTA in the same sections. **(B)** Scale bars = 100 um; **(C)** scale bars = 20 um. **(D,E)** High power images of visual cortex and entorhinal cortex in the same section. **(D)** Scale bar = 100 um. **(E)** Scale bars = 20 um.

Supplementary Figure 13 Qualitative images of CD68 staining in C57BL6 and CX3CR1^+/GFP^ treated with 5 mg/kg of LPS and relative vehicles. This panel compares the increase of CD68 in SNpr, thalamus, hypothalamus and PSM cortex. It is possible to appreciate the absence or lower activation state in CX3CR1^+/GFP^ mice treated with LPS compared with the same group in C57BL6 mice. Scale bars = 40 um.

Supplementary Figure 14 **(A)** Qualitative images of GFAP staining in C57BL6 mice treated with 5 mg/kg of LPS and relative vehicles. It is possible to appreciate activation state in the corpus callosum of these mice. **(B)** Qualitative images of GFAP staining with different magnification in C57BL6 mice treated with 5 mg/kg of LPS and relative vehicles. The peculiar morphology observed in cerebellum with the appearance of astrocytes in the cortical region of the simple lobule. Particular examples were showed in the insets. **(C)** Qualitative images about the absence of astrocytic activation in SNpr in CX3CR1^+/GFP^ mice treated with LPS and relative vehicle.

## Abbreviations

AD: Alzheimer’s disease
BBB: blood-brain barrier
CD68: cluster of differentiation 68
CNS: central nervous system
CVO: circumventricular organ
EFI: extended focus imaging
GABA: gamma-aminobutyric acid
GFAP: glial fibrillary acid protein
IL: interleukin
i.p.: intraperitoneal
LPS: lipopolysaccharide
MHC: major histocompatibility complex
MPTP: 1-methyl-4-phenyl-1,2,3,6-tetrahydropyridine
NAc: nucleus accumbens
NLPR3: Nucleotide-binding domain and leucine-rich repeat receptor containing a pyrin domain 3
PD: Parkinson’s disease
PFA: paraformaldehyde
PPA cortex: posterior parietal association areas
PSM cortex: primary motor and somatosensory cortex
ROS: reactive oxygen species
SNpr: substantia nigra pars reticulate
SNpc: substantia nigra part compacta
TGF: transforming growth factor
TH: tyrosine hydroxylase
TLR: toll-like receptors
TNF: tumor necrosis factor
VTA: ventral tegmental area
6-OHDA: 6-hydroxydopamine.
