# Supplementary material for: Brain region-specific microglial and astrocytic activation in response to systemic lipopolysaccharides exposure
Source: Front Aging Neurosci. 2022 Aug 26;14:910988. doi: 10.3389/fnagi.2022.910988 (PMC9459169; doi:10.3389/fnagi.2022.910988)
Supplement: Supplementary file 1 [file Data_Sheet_1.PDF]

Supplementary table 1

Tukey's multiple comparisons test	Mean Diff,	95,00% CI of diff,	Summary	Adjusted P Value
NAc vs. VTA	2,513	0,4526 to 4,574	**	0,0064
NAc vs. SNpr	-2,62	-4,681 to -0,5593	**	0,0038
NAc vs. Cerebellum	2,503	0,4426 to 4,564	**	0,0067
NAc vs. Corpus Callosum	2,157	0,09594 to 4,217	*	0,0331
Striatum vs. VTA	2,907	0,8459 to 4,967	***	0,0009
Striatum vs. SNpr	-2,227	-4,287 to -0,1659	*	0,0242
Striatum vs. Cerebellum	2,897	0,8359 to 4,957	***	0,001
Striatum vs. Corpus Callosum	2,55	0,4893 to 4,611	**	0,0054
Thalamus vs. SNpr	-4,177	-6,237 to -2,116	****	<0,0001
Thalamus vs. Piriform Cortex	-2,117	-4,177 to -0,05594	*	0,0394
Hypothalamus vs. SNpr	-4,06	-6,121 to -1,999	****	<0,0001
Amygdala vs. VTA	2,197	0,1359 to 4,257	*	0,0277
Amygdala vs. SNpr	-2,937	-4,997 to -0,8759	***	0,0008
Amygdala vs. Cerebellum	2,187	0,1259 to 4,247	*	0,029
VTA vs. SNpr	-5,133	-7,194 to -3,073	****	<0,0001
VTA vs. PSM Cortex	-2,853	-4,914 to -0,7926	**	0,0012
VTA vs. PPA Cortex	-2,967	-5,027 to -0,9059	***	0,0007
VTA vs. Visual Cortex	-2,767	-4,827 to -0,7059	**	0,0019
VTA vs. Piriform Cortex	-3,073	-5,134 to -1,013	***	0,0004
VTA vs. Entorhinal Cortex	-2,767	-4,827 to -0,7059	**	0,0019
VTA vs. Hippocampus	-2,31	-4,371 to -0,2493	*	0,0166
SNpc vs. SNpr	-3,767	-5,827 to -1,706	****	<0,0001
SNpr vs. PSM Cortex	2,28	0,2193 to 4,341	*	0,019
SNpr vs. PPA Cortex	2,167	0,1059 to 4,227	*	0,0316
SNpr vs. Visual Cortex	2,367	0,3059 to 4,427	*	0,0128
SNpr vs. Entorhinal Cortex	2,367	0,3059 to 4,427	*	0,0128
SNpr vs. Hippocampus	2,823	0,7626 to 4,884	**	0,0014
SNpr vs. Cerebellum	5,123	3,063 to 7,184	****	<0,0001
SNpr vs. Corpus Callosum	4,777	2,716 to 6,837	****	<0,0001
PSM Cortex vs. Cerebellum	2,843	0,7826 to 4,904	**	0,0013
PSM Cortex vs. Corpus Callosum	2,497	0,4359 to 4,557	**	0,0069
PPA Cortex vs. Cerebellum	2,957	0,8959 to 5,017	***	0,0007
PPA Cortex vs. Corpus Callosum	2,61	0,5493 to 4,671	**	0,004
Visual Cortex vs. Cerebellum	2,757	0,6959 to 4,817	**	0,0019
Visual Cortex vs. Corpus Callosum	2,41	0,3493 to 4,471	*	0,0104
Piriform Cortex vs. Cerebellum	3,063	1,003 to 5,124	***	0,0004
Piriform Cortex vs. Corpus Callosum	2,717	0,6559 to 4,777	**	0,0024
Entorhinal Cortex vs. Cerebellum	2,757	0,6959 to 4,817	**	0,0019
Entorhinal Cortex vs. Corpus Callosum	2,41	0,3493 to 4,471	*	0,0104
Hippocampus vs. Cerebellum	2,3	0,2393 to 4,361	*	0,0174

Supplementary table 2

Tukey's multiple comparisons test	Mean Diff,	95,00% CI of diff,	Summary	Adjusted P Value
NAc vs. Thalamus	94,33	8,546 to 180,1	*	0,0203
NAc vs. Hypothalamus	91	5,213 to 176,8	*	0,0291
NAc vs. VTA	144,3	58,55 to 230,1	****	<0,0001
NAc vs. SNpr	-187,7	-273,5 to -101,9	****	<0,0001
NAc vs. Cerebellum	158	72,21 to 243,8	****	<0,0001
NAc vs. Corpus Callosum	140,7	54,88 to 226,5	****	<0,0001
Striatum vs. Thalamus	100	14,21 to 185,8	*	0,0108
Striatum vs. Hypothalamus	96,67	10,88 to 182,5	*	0,0157
Striatum vs. VTA	150	64,21 to 235,8	****	<0,0001
Striatum vs. SNpr	-182,1	-267,9 to -96,28	****	<0,0001
Striatum vs. Cerebellum	163,7	77,88 to 249,5	****	<0,0001
Striatum vs. Corpus Callosum	146,3	60,55 to 232,1	****	<0,0001
Thalamus vs. Amygdala	-101,3	-187,1 to -15,55	**	0,0093
Thalamus vs. SNpr	-282,1	-367,9 to -196,3	****	<0,0001
Thalamus vs. PSM Cortex	-131	-216,8 to -45,21	***	0,0003
Thalamus vs. PPA Cortex	-132,3	-218,1 to -46,55	***	0,0002
Thalamus vs. Visual Cortex	-133,7	-219,5 to -47,88	***	0,0002
Thalamus vs. Piriform Cortex	-103,7	-189,5 to -17,88	**	0,0071
Thalamus vs. Entorhinal Cortex	-104	-189,8 to -18,21	**	0,0069
Hypothalamus vs. Amygdala	-98	-183,8 to -12,21	*	0,0135
Hypothalamus vs. SNpr	-278,7	-364,5 to -192,9	****	<0,0001
Hypothalamus vs. PSM Cortex	-127,7	-213,5 to -41,88	***	0,0004
Hypothalamus vs. PPA Cortex	-129	-214,8 to -43,21	***	0,0004
Hypothalamus vs. Visual Cortex	-130,3	-216,1 to -44,55	***	0,0003
Hypothalamus vs. Piriform Cortex	-100,3	-186,1 to -14,55	*	0,0104
Hypothalamus vs. Entorhinal Cortex	-100,7	-186,5 to -14,88	*	0,01
Amygdala vs. VTA	151,3	65,55 to 237,1	****	<0,0001
Amygdala vs. SNpr	-180,7	-266,5 to -94,95	****	<0,0001
Amygdala vs. Cerebellum	165	79,21 to 250,8	****	<0,0001
Amygdala vs. Corpus Callosum	147,7	61,88 to 233,5	****	<0,0001
VTA vs. SNpc	-86,67	-172,5 to -0,8794	*	0,0457
VTA vs. SNpr	-332,1	-417,9 to -246,3	****	<0,0001
VTA vs. PSM Cortex	-181	-266,8 to -95,21	****	<0,0001
VTA vs. PPA Cortex	-182,3	-268,1 to -96,55	****	<0,0001
VTA vs. Visual Cortex	-183,7	-269,5 to -97,88	****	<0,0001
VTA vs. Piriform Cortex	-153,7	-239,5 to -67,88	****	<0,0001
VTA vs. Entorhinal Cortex	-154	-239,8 to -68,21	****	<0,0001
VTA vs. Hippocampus	-134,3	-220,1 to -48,55	***	0,0002
SNpc vs. SNpr	-245,4	-331,2 to -159,6	****	<0,0001
SNpc vs. PSM Cortex	-94,33	-180,1 to -8,546	*	0,0203
SNpc vs. PPA Cortex	-95,67	-181,5 to -9,879	*	0,0175
SNpc vs. Visual Cortex	-97	-182,8 to -11,21	*	0,0151
SNpc vs. Cerebellum	100,3	14,55 to 186,1	*	0,0104
SNpr vs. PSM Cortex	151,1	65,28 to 236,9	****	<0,0001
SNpr vs. PPA Cortex	149,7	63,95 to 235,5	****	<0,0001
SNpr vs. Visual Cortex	148,4	62,61 to 234,2	****	<0,0001
SNpr vs. Piriform Cortex	178,4	92,61 to 264,2	****	<0,0001
SNpr vs. Entorhinal Cortex	178,1	92,28 to 263,9	****	<0,0001
SNpr vs. Hippocampus	197,7	111,9 to 283,5	****	<0,0001
SNpr vs. Cerebellum	345,7	259,9 to 431,5	****	<0,0001
SNpr vs. Corpus Callosum	328,4	242,6 to 414,2	****	<0,0001
PSM Cortex vs. Cerebellum	194,7	108,9 to 280,5	****	<0,0001
PSM Cortex vs. Corpus Callosum	177,3	91,55 to 263,1	****	<0,0001
PPA Cortex vs. Cerebellum	196	110,2 to 281,8	****	<0,0001
PPA Cortex vs. Corpus Callosum	178,7	92,88 to 264,5	****	<0,0001
Visual Cortex vs. Cerebellum	197,3	111,5 to 283,1	****	<0,0001
Visual Cortex vs. Corpus Callosum	180	94,21 to 265,8	****	<0,0001
Piriform Cortex vs. Cerebellum	167,3	81,55 to 253,1	****	<0,0001
Piriform Cortex vs. Corpus Callosum	150	64,21 to 235,8	****	<0,0001
Entorhinal Cortex vs. Cerebellum	167,7	81,88 to 253,5	****	<0,0001
Entorhinal Cortex vs. Corpus Callosum	150,3	64,55 to 236,1	****	<0,0001
Hippocampus vs. Cerebellum	148	62,21 to 233,8	****	<0,0001
Hippocampus vs. Corpus Callosum	130,7	44,88 to 216,5	***	0,0003

Supplementary table 3

Tukey's multiple comparisons test	Mean Diff,	95,00% CI of diff,	Summary	Adjusted P Value
**NAc vs. VTA**	91,67	18,46 to 164,9	**	0,0046
**NAc vs. SNpc**	93	19,79 to 166,2	**	0,0039
**NAc vs. Cerebellum**	100,7	27,46 to 173,9	**	0,0013
**Striatum vs. VTA**	75,33	2,127 to 148,5	*	0,0387
**Striatum vs. SNpc**	76,67	3,460 to 149,9	*	0,0328
**Striatum vs. Entorhinal Cortex**	-78,67	-151,9 to -5,460	*	0,0256
**Striatum vs. Cerebellum**	84,33	11,13 to 157,5	*	0,0123
**Thalamus vs. Entorhinal Cortex**	-82,67	-155,9 to -9,460	*	0,0153
**Thalamus vs. Cerebellum**	80,33	7,127 to 153,5	*	0,0207
**Hypothalamus vs. VTA**	100	26,79 to 173,2	**	0,0015
**Hypothalamus vs. SNpc**	101,3	28,13 to 174,5	**	0,0012
**Hypothalamus vs. Cerebellum**	109	35,79 to 182,2	***	0,0004
**Amygdala vs. VTA**	90	16,79 to 163,2	**	0,0058
**Amygdala vs. SNpc**	91,33	18,13 to 164,5	**	0,0048
**Amygdala vs. Cerebellum**	99	25,79 to 172,2	**	0,0017
**VTA vs. SNpr**	-73,33	-146,5 to -0,1267	*	0,0493
**VTA vs. PSM Cortex**	-102,3	-175,5 to -29,13	**	0,0011
**VTA vs. PPA Cortex**	-130,7	-203,9 to -57,46	****	<0,0001
**VTA vs. Visual Cortex**	-105,3	-178,5 to -32,13	***	0,0007
**VTA vs. Piriform Cortex**	-137	-210,2 to -63,79	****	<0,0001
**VTA vs. Entorhinal Cortex**	-154	-227,2 to -80,79	****	<0,0001
**VTA vs. Hippocampus**	-112	-185,2 to -38,79	***	0,0003
**SNpc vs. SNpr**	-74,67	-147,9 to -1,460	*	0,042
**SNpc vs. PSM Cortex**	-103,7	-176,9 to -30,46	***	0,0009
**SNpc vs. PPA Cortex**	-132	-205,2 to -58,79	****	<0,0001
**SNpc vs. Visual Cortex**	-106,7	-179,9 to -33,46	***	0,0006
**SNpc vs. Piriform Cortex**	-138,3	-211,5 to -65,13	****	<0,0001
**SNpc vs. Entorhinal Cortex**	-155,3	-228,5 to -82,13	****	<0,0001
**SNpc vs. Hippocampus**	-113,3	-186,5 to -40,13	***	0,0002
**SNpr vs. Entorhinal Cortex**	-80,67	-153,9 to -7,460	*	0,0199
**SNpr vs. Cerebellum**	82,33	9,127 to 155,5	*	0,016
**PSM Cortex vs. Cerebellum**	111,3	38,13 to 184,5	***	0,0003
**PPA Cortex vs. Cerebellum**	139,7	66,46 to 212,9	****	<0,0001
**PPA Cortex vs. Corpus Callosum**	93,33	20,13 to 166,5	**	0,0037
**Visual Cortex vs. Cerebellum**	114,3	41,13 to 187,5	***	0,0002
**Piriform Cortex vs. Cerebellum**	146	72,79 to 219,2	****	<0,0001
**Piriform Cortex vs. Corpus Callosum**	99,67	26,46 to 172,9	**	0,0015
**Entorhinal Cortex vs. Cerebellum**	163	89,79 to 236,2	****	<0,0001
**Entorhinal Cortex vs. Corpus Callosum**	116,7	43,46 to 189,9	***	0,0001
**Hippocampus vs. Cerebellum**	121	47,79 to 194,2	****	<0,0001
**Hippocampus vs. Corpus Callosum**	74,67	1,460 to 147,9	*	0,042

Supplementary table 4A

Tukey's multiple comparisons test	Mean Diff,	95,00% CI of diff,	Summary	Adjusted P Value
**NAc vs. VTA**	-0,9467	-1,567 to -0,3266	***	0,0003
**Striatum vs. VTA**	-1,037	-1,657 to -0,4166	****	<0,0001
**Thalamus vs. PSM Cortex**	0,8367	0,2166 to 1,457	**	0,0017
**Hypothalamus vs. VTA**	-0,7267	-1,347 to -0,1066	*	0,0102
**Hypothalamus vs. PSM Cortex**	0,67	0,04997 to 1,290	*	0,0242
**Amygdala vs. VTA**	-0,9467	-1,567 to -0,3266	***	0,0003
**VTA vs. SNpr**	1,107	0,4866 to 1,727	****	<0,0001
**VTA vs. PSM Cortex**	1,397	0,7766 to 2,017	****	<0,0001
**VTA vs. PPA Cortex**	1,16	0,5400 to 1,780	****	<0,0001
**VTA vs. Visual Cortex**	1,08	0,4600 to 1,700	****	<0,0001
**VTA vs. Piriform Cortex**	1,02	0,4000 to 1,640	****	<0,0001
**VTA vs. Entorhinal Cortex**	0,7333	0,1133 to 1,353	**	0,0092
**VTA vs. Hippocampus**	0,7333	0,1133 to 1,353	**	0,0092
**VTA vs. Cerebellum**	0,96	0,3400 to 1,580	***	0,0002
**VTA vs. Corpus Callosum**	0,83	0,2100 to 1,450	**	0,0019
**SNpc vs. PSM Cortex**	0,8733	0,2533 to 1,493	***	0,0009
**SNpc vs. PPA Cortex**	0,6367	0,01663 to 1,257	*	0,0395
**PSM Cortex vs. Entorhinal Cortex**	-0,6633	-1,283 to -0,04330	*	0,0267
**PSM Cortex vs. Hippocampus**	-0,6633	-1,283 to -0,04330	*	0,0267

Supplementary table 4B

Tukey's multiple comparisons test	Mean Diff,	95,00% CI of diff,	Summary	Adjusted P Value
**Amygdala vs. Entorhinal Cortex**	-0,6367	-1,196 to -0,07708	*	0,0141
**VTA vs. SNpr**	0,58	0,02042 to 1,140	*	0,0362
**VTA vs. PSM Cortex**	0,7367	0,1771 to 1,296	**	0,0024
**SNpc vs. SNpr**	0,5867	0,02708 to 1,146	*	0,0325
**SNpc vs. PSM Cortex**	0,7433	0,1837 to 1,303	**	0,0021
**SNpr vs. Entorhinal Cortex**	-0,7333	-1,293 to -0,1737	**	0,0026
**PSM Cortex vs. Entorhinal Cortex**	-0,89	-1,450 to -0,3304	***	0,0001
**PSM Cortex vs. Corpus Callosum**	-0,6467	-1,206 to -0,08708	*	0,0119
**PPA Cortex vs. Entorhinal Cortex**	-0,64	-1,200 to -0,08042	*	0,0134
**Visual Cortex vs. Entorhinal Cortex**	-0,6267	-1,186 to -0,06708	*	0,0168

Supplementary table 5

Tukey's multiple comparisons test	Mean Diff,	95,00% CI of diff,	Summary	Adjusted P Value
NAc vs. Hypothalamus	-4,833	-7,585 to -2,081	****	<0,0001
NAc vs. Amygdala	-3,133	-5,885 to -0,3813	*	0,014
NAc vs. SNpc	-4,9	-7,652 to -2,148	****	<0,0001
NAc vs. Entorhinal Cortex	-5,3	-8,052 to -2,548	****	<0,0001
Striatum vs. Hypothalamus	-4,467	-7,219 to -1,715	***	0,0001
Striatum vs. Amygdala	-2,767	-5,519 to -0,01459	*	0,0477
Striatum vs. SNpc	-4,533	-7,285 to -1,781	****	<0,0001
Striatum vs. Entorhinal Cortex	-4,933	-7,685 to -2,181	****	<0,0001
Thalamus vs. Hypothalamus	-4,733	-7,485 to -1,981	****	<0,0001
Thalamus vs. Amygdala	-3,033	-5,785 to -0,2813	*	0,0198
Thalamus vs. SNpc	-4,8	-7,552 to -2,048	****	<0,0001
Thalamus vs. Entorhinal Cortex	-5,2	-7,952 to -2,448	****	<0,0001
Hypothalamus vs. VTA	3,833	1,081 to 6,585	**	0,0011
Hypothalamus vs. PSM Cortex	4,467	1,715 to 7,219	***	0,0001
Hypothalamus vs. PPA Cortex	4	1,248 to 6,752	***	0,0006
Hypothalamus vs. Visual Cortex	4,4	1,648 to 7,152	***	0,0001
Hypothalamus vs. Cerebellum	3,1	0,3479 to 5,852	*	0,0158
Amygdala vs. PSM Cortex	2,767	0,01459 to 5,519	*	0,0477
VTA vs. SNpc	-3,9	-6,652 to -1,148	***	0,0009
VTA vs. Entorhinal Cortex	-4,3	-7,052 to -1,548	***	0,0002
VTA vs. Cerebellum	-0,7333	-3,485 to 2,019	ns	0,9996
SNpc vs. PSM Cortex	4,533	1,781 to 7,285	****	<0,0001
SNpc vs. PPA Cortex	4,067	1,315 to 6,819	***	0,0005
SNpc vs. Visual Cortex	4,467	1,715 to 7,219	***	0,0001
SNpc vs. Cerebellum	3,167	0,4146 to 5,919	*	0,0125
PSM Cortex vs. Entorhinal Cortex	-4,933	-7,685 to -2,181	****	<0,0001
PPA Cortex vs. Entorhinal Cortex	-4,467	-7,219 to -1,715	***	0,0001
Visual Cortex vs. Entorhinal Cortex	-4,867	-7,619 to -2,115	****	<0,0001
Entorhinal Cortex vs. Cerebellum	3,567	0,8146 to 6,319	**	0,003
Hippocampus vs. Cerebellum	16,77	14,01 to 19,52	****	<0,0001

Supplementary table 6

Tukey's multiple comparisons test	Mean Diff,	95,00% CI of diff,	Summary	Adjusted P Value
NAc vs. Corpus Callosum	4,263	0,2313 to 8,295	*	0,03
Striatum vs. Corpus Callosum	5,183	1,151 to 9,215	**	0,0033
Thalamus vs. Hypothalamus	5,01	0,9779 to 9,042	**	0,0051
Thalamus vs. Amygdala	5,623	1,591 to 9,655	**	0,0011
Thalamus vs. VTA	4,56	0,5279 to 8,592	*	0,0151
Thalamus vs. SNpc	4,047	0,01461 to 8,079	*	0,0484
Thalamus vs. Piriform Cortex	6,717	2,685 to 10,75	****	<0,0001
Thalamus vs. Enthorinal Cortex	5,553	1,521 to 9,585	**	0,0013
Thalamus vs. Hippocampus	5,907	1,875 to 9,939	***	0,0005
Thalamus vs. Corpus Callosum	8,563	4,531 to 12,60	****	<0,0001
Hypothalamus vs. PSM Cortex	-4,28	-8,312 to -0,2479	*	0,0289
Amygdala vs. PSM Cortex	-4,893	-8,925 to -0,8613	**	0,0068
Amygdala vs. Visual Cortex	-4,24	-8,272 to -0,2079	*	0,0316
SNpc vs. Corpus Callosum	4,517	0,4846 to 8,549	*	0,0167
SNpr vs. Corpus Callosum	5,37	1,338 to 9,402	**	0,0021
PSM Cortex vs. Piriform Cortex	5,987	1,955 to 10,02	***	0,0004
PSM Cortex vs. Enthorinal Cortex	4,823	0,7913 to 8,855	**	0,008
PSM Cortex vs. Hippocampus	5,177	1,145 to 9,209	**	0,0034
PSM Cortex vs. Corpus Callosum	7,833	3,801 to 11,87	****	<0,0001
PPA Cortex vs. Piriform Cortex	4,983	0,9513 to 9,015	**	0,0054
PPA Cortex vs. Hippocampus	4,173	0,1413 to 8,205	*	0,0367
PPA Cortex vs. Corpus Callosum	6,83	2,798 to 10,86	****	<0,0001
Visual Cortex vs. Piriform Cortex	5,333	1,301 to 9,365	**	0,0023
Visual Cortex vs. Enthorinal Cortex	4,17	0,1379 to 8,202	*	0,0369
Visual Cortex vs. Hippocampus	4,523	0,4913 to 8,555	*	0,0165
Visual Cortex vs. Corpus Callosum	7,18	3,148 to 11,21	****	<0,0001
Hippocampus vs. Corpus Callosum	2,657	-1,375 to 6,689	ns	0,5321
