# Supplementary figures and images for: Brain region-specific microglial and astrocytic activation in response to systemic lipopolysaccharides exposure
Source: Front Aging Neurosci. 2022 Aug 26;14:910988. doi: 10.3389/fnagi.2022.910988 (PMC9459169; doi:10.3389/fnagi.2022.910988)

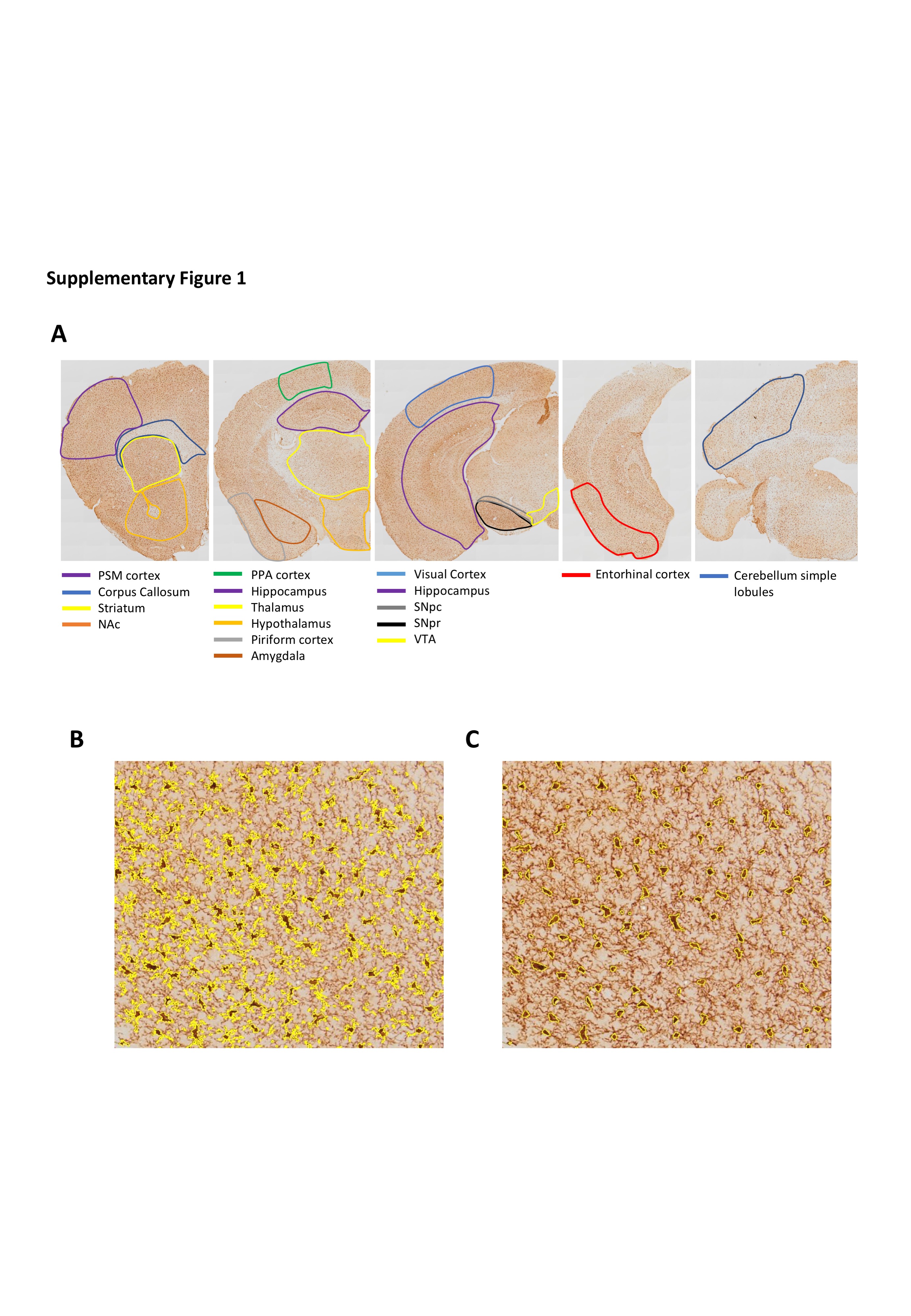

Supplement: Supplementary Figure 1 — (A) Qualitative examples about ROI sampling in some sections. The legend for colors is located under the respective images. The scanned sections are labeled with Iba1. (B) Qualitative images about the microglial marked area analyses. The image show outlines around soma and main branches. Section stained with Iba1. (C) Qualitative images about the microglial density analyses. The image show outlines exclusively around the soma. Section stained with Iba1. [file Image_1.JPEG]

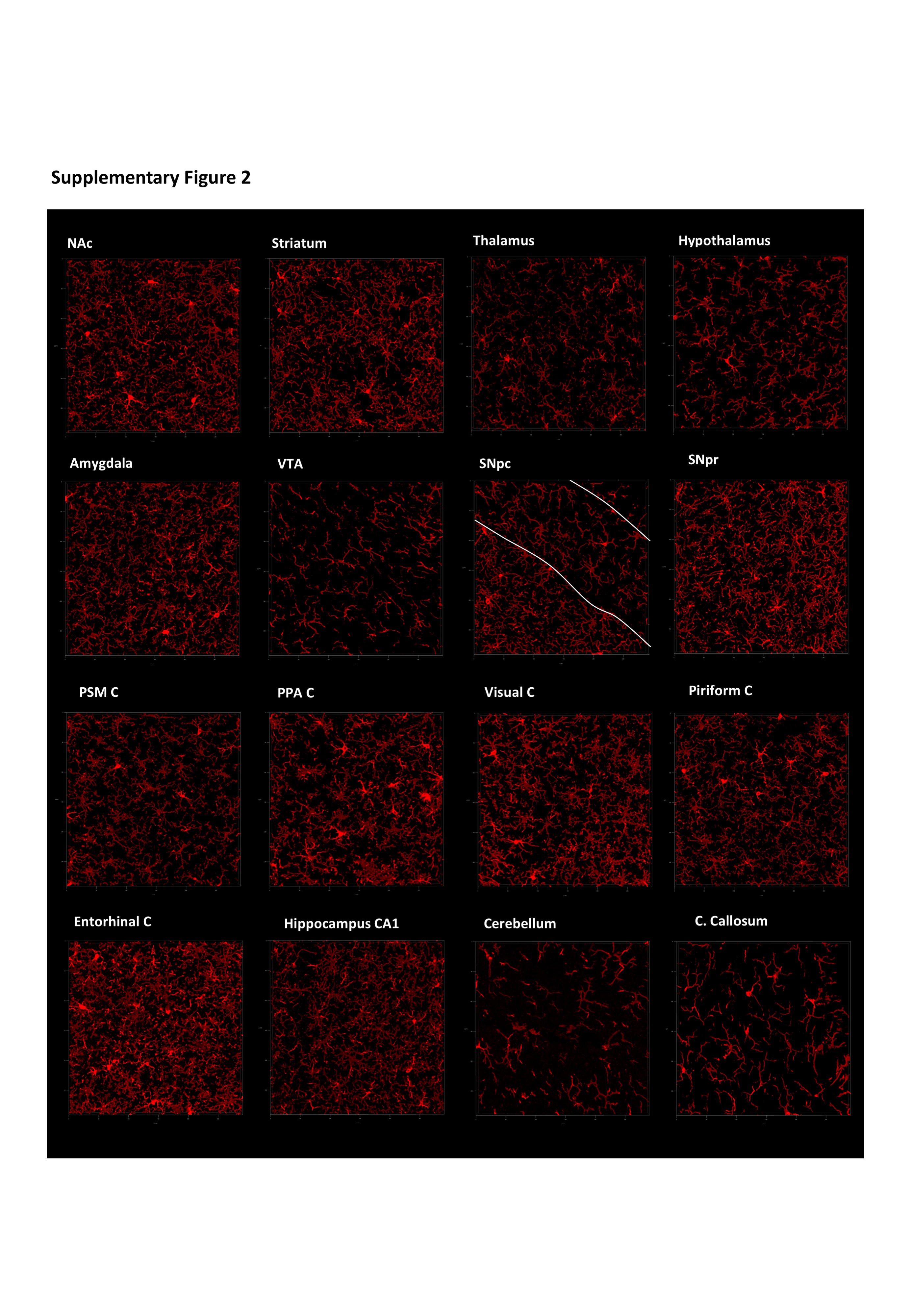

Supplement: Supplementary Figure 2 — 3D confocal images of microglial cells in all 16 regions examined. Sections labeled with Iba1-Cy3 and acquired with 63x magnification with z-stack of 11 um with 0.5 um increment between optical sections. Scale bar = 50 um. [file Image_2.JPEG]

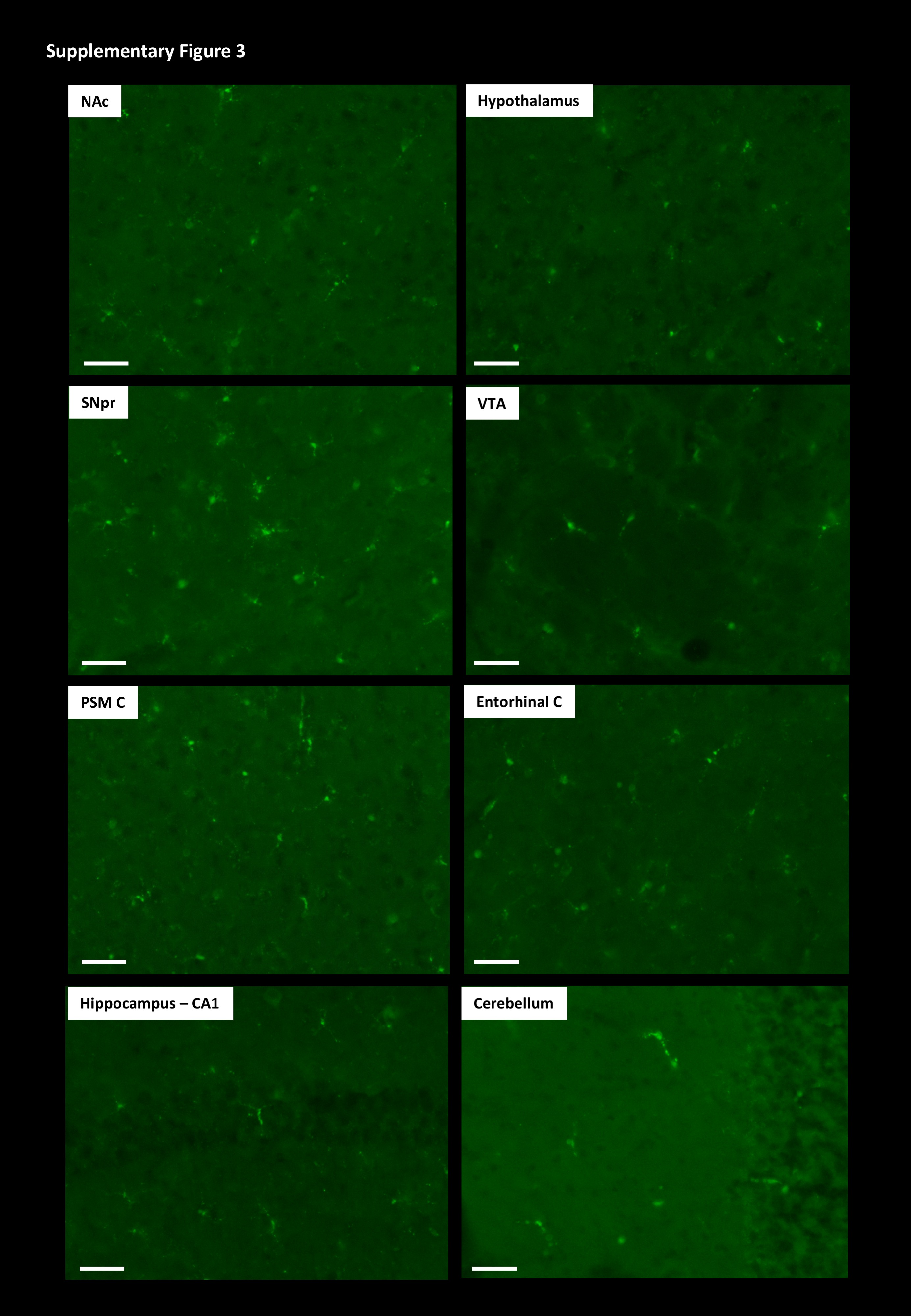

Supplement: Supplementary Figure 3 — Qualitative images of CD68 staining in different brain regions in control C57BL6 mice. This panel shows the different CD68 distribution in NAc, hypothalamus, SNpr, VTA, PSM cortex, entorhinal cortex, hippocampus, and cerebellum. It is possible to appreciate the higher amount of vesicle in SNpr in respect to all other regions. Scale bars = 20 um. [file Image_3.jpg]

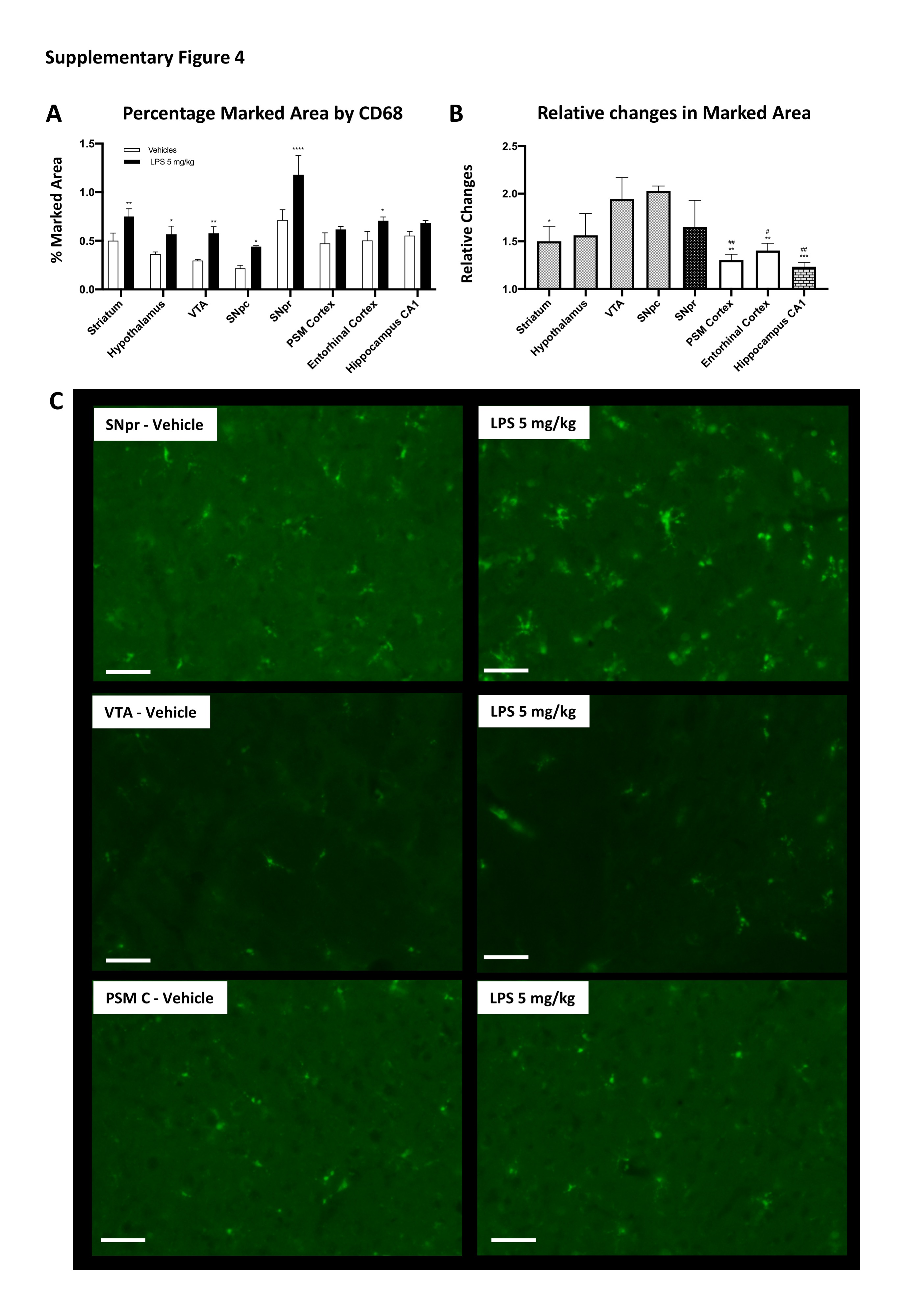

Supplement: Supplementary Figure 4 — Alterations of CD68 positive cells in response to the treatment of LPS. (A) Change in percentage marked area by CD68 immunoreactivity among different brain regions. Two-way ANOVA. FBrain Regions (7, 32) = 32.12, p < 0.0001. FTreatment (1, 32) = 107.3, p < 0.0001. FInteraction (7, 32) = 2.631, p = 0.0289, and n = 3 mice per region. Significance: (*) respect relative vehicles. (B) The relative changes in the marked areas between the vehicle- and LPS 5mg/kg treatments among different brain regions. One way ANOVA FBrain Regions (7, 16) = 8.982, p = 0.0002, and n = 3 mice per region. Significance: (*) respect SNpc; (#) respect VTA. (C) Representative images of CD68 staining in different brain regions (SNpr, VTA, and PSM cortex) in vehicle- and LPS (5 mg/kg) treated C57BL6 mice. It is evident that CD68 immunoreactivity is higher in SNpr compared to other brain regions. Scale bars = 20 um. [file Image_4.JPEG]

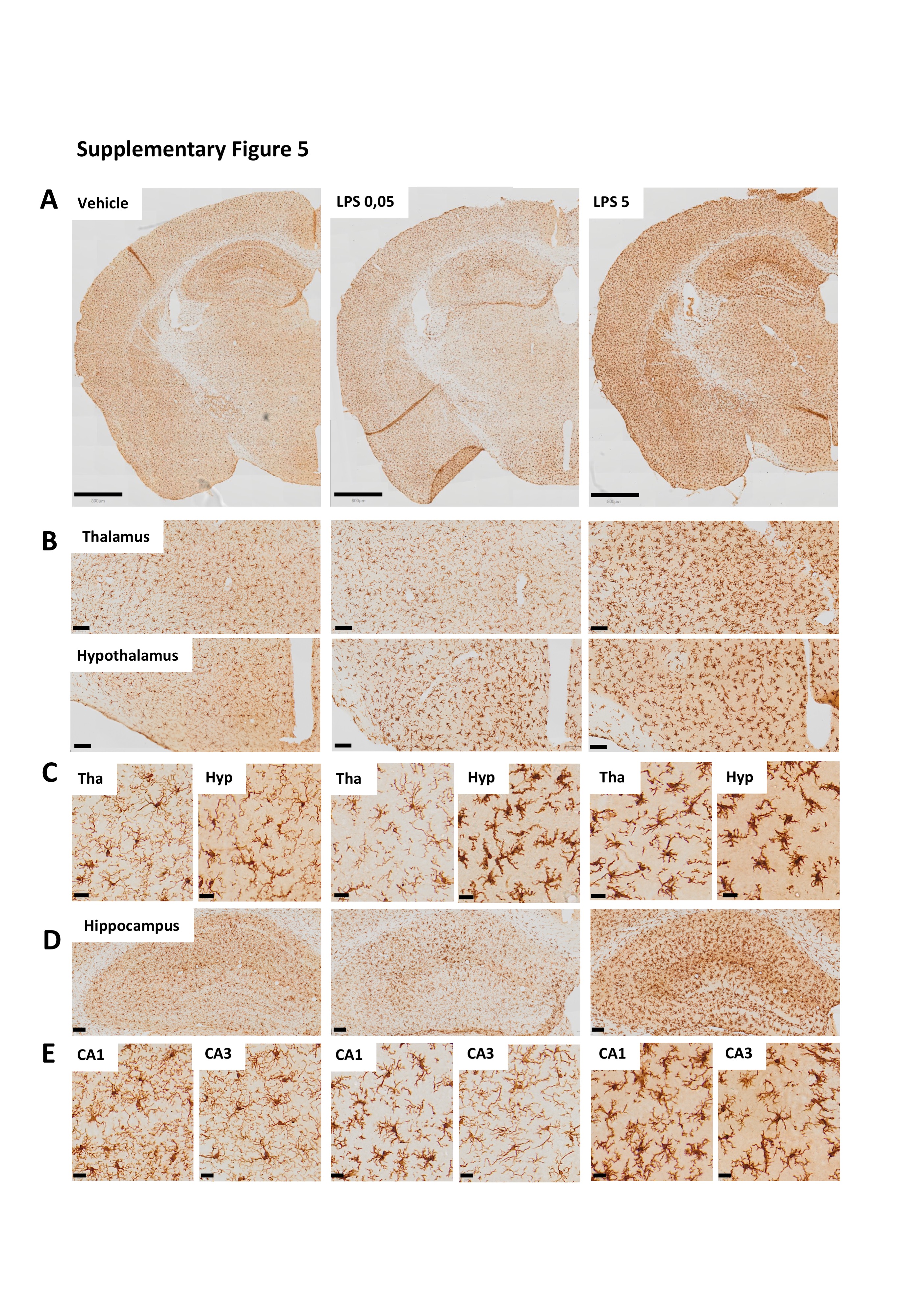

Supplement: Supplementary Figure 5 — Qualitative images of dose-dependent microglial activation in mice treated with 0.05 and 5 mg/kg of LPS and relative vehicles. This panel of images shows the different microglial sensitivity and activation in the hypothalamus compared with all other regions in the same section. Moreover, panels (D,E) represent good examples of intra-regional differences. Microglia activations within the hippocampus seem to be more sensitive in CA1 and subiculum respect to CA3 and gyrus dentatus. (A) Scale bar = 800 um. (B,C) High power images of the thalamus and hypothalamus. (B) Scale bar = 100 um; (C) scale bar = 20 um. (D,E) High power images of the hippocampus in the same section. (D) Scale bar = 100 um; (E) high power images of the CA1 and CA3. Scale bar = 20 um. [file Image_5.JPEG]

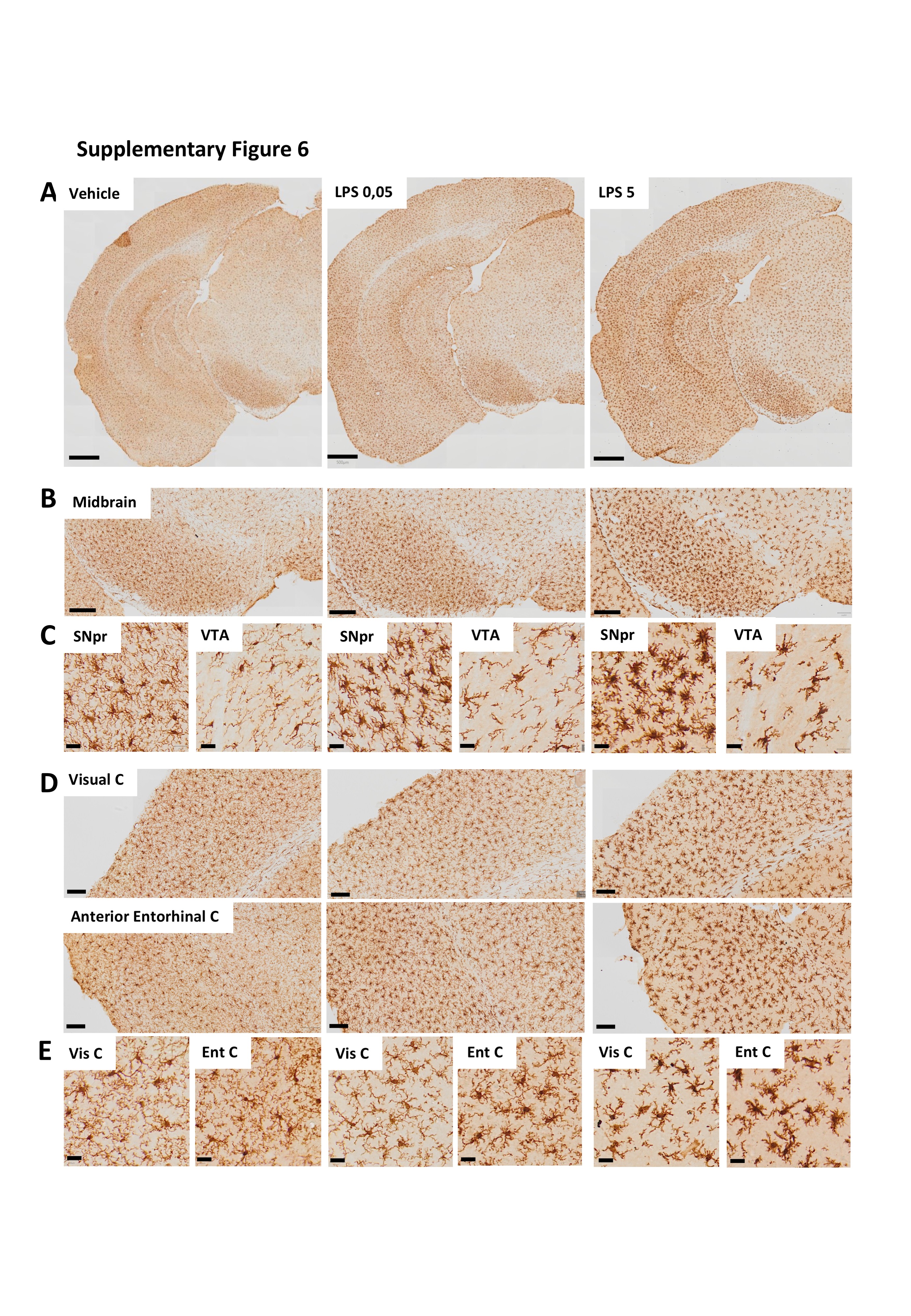

Supplement: Supplementary Figure 6 — Qualitative images of dose-dependent microglial activation in mice treated with 0.05 and 5 mg/kg of LPS and relative vehicles. This panel of images shows the different microglial sensitivity between SNpr, VTA, and anterior entorhinal cortex with respect to visual cortex. (A) Scale bar = 500 um. (B,C) High power images of SNpr and VTA in the same section. (B) Scale bar = 200 um; (C) scale = bar 20 um. (D,E) High power images of visual and entorhinal cortex in the same section. (D) Scale bar = 100 um. (E) Scale bar = 20 um. [file Image_6.JPEG]

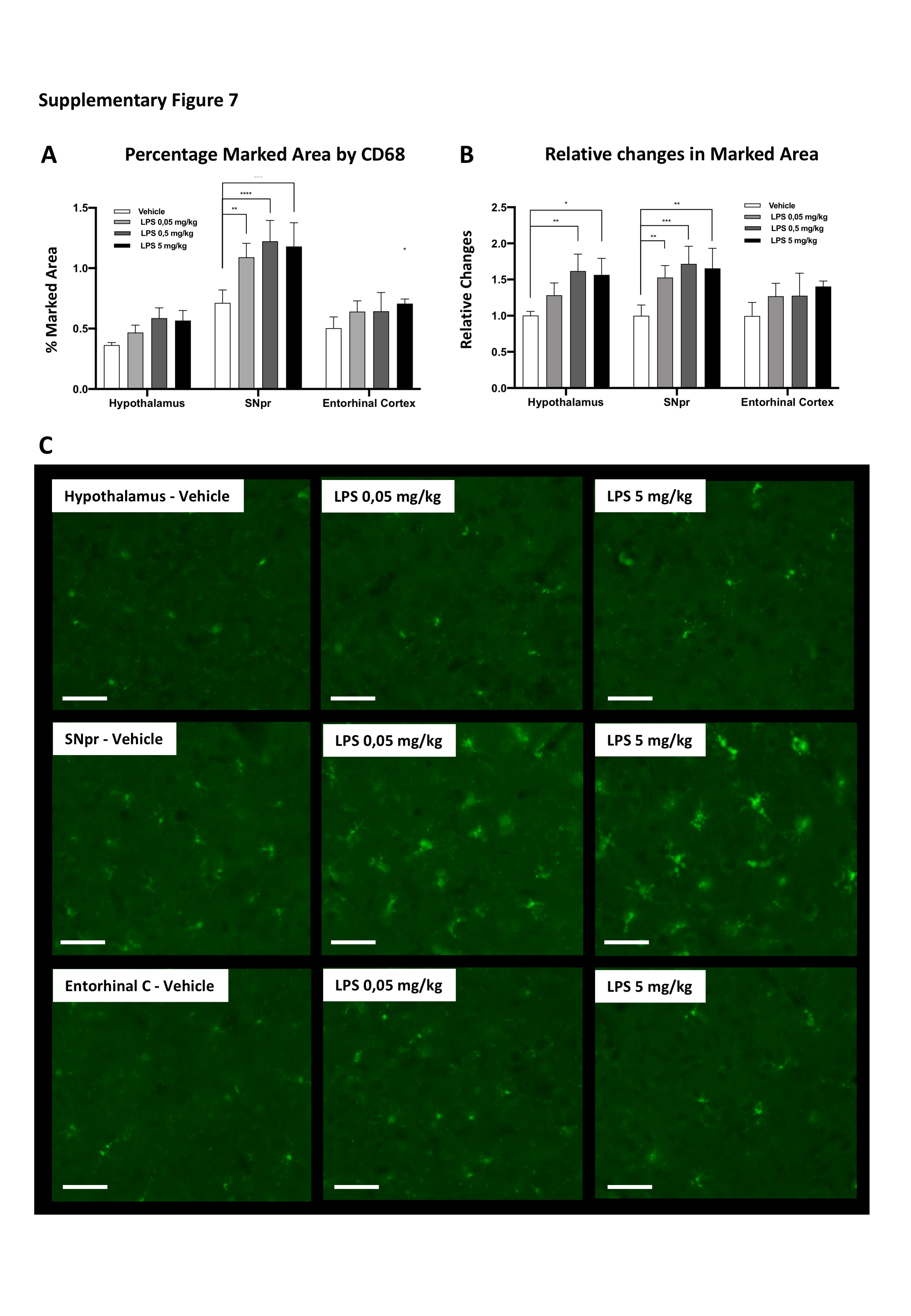

Supplement: Supplementary Figure 7 — Alterations of CD68 in response to different doses of LPS treatments. (A) Change in percentage marked area by CD68 immunoreactivity in vehicles (white columns), LPS treated mice with 0.05 mg/kg (medium gray columns), 0.5 mg/kg (dark gray columns), 5 mg/kg (black columns). Two-way ANOVA. FBrain Regions (2, 24) = 79.43, p < 0.0001. FTreatment (3, 24) = 13.26, p < 0.0001. FInteraction (6, 24) = 1.787, p < 0.1443, and n = 3 mice per region. Significance: respect relative vehicles. (B) The relative changes in the marked areas between the vehicle- and different doses of LPS (0.05-5mg/kg) treatments among different brain regions. The same column color as graph (A). Two-way ANOVA with Tukey correction. FBrain Regions (2, 24) = 4.131, p < 0.0001. FTreatment (3, 24) = 14.11, p < 0.0287. FInteraction (6, 24) = 0.8165, p < 0,5678, and n = 3 mice per region. Significance: (*) respect relative vehicles. (C) Representative images of CD68 staining in the dose-dependent in mice treated with 0.05 mg/kg and 5 mg/kg of LPS and relative vehicles. This panel shows the increase of CD68 in hypothalamus, SNpr and entorhinal cortex. CD68 immunoreactivity appears more robust in the low dose LPS exposure in SNpr compared to other brain regions. Scale bars = 20 um. [file Image_7.JPEG]

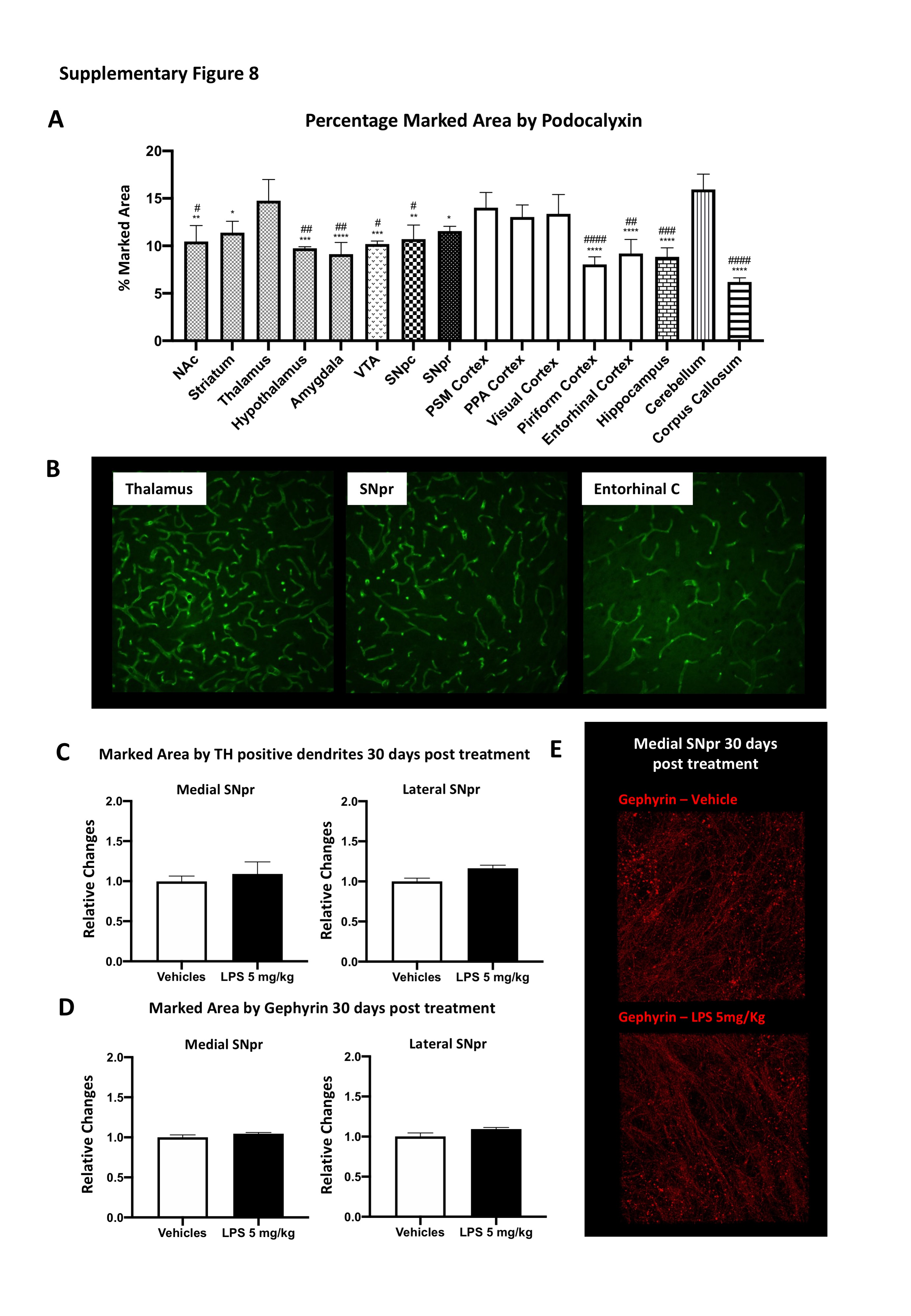

Supplement: Supplementary Figure 8 — (A) Percentage of the marked area by podocalyxin in vehicles C57BL6N mice. One-way ANOVA FBrain Regions (15, 32) = 11.69, p < 0.0001, and n = 3 mice per region. Significance: (*) respect the cerebellum; (#) respect the thalamus. Other significances are summarized in Supplementary Table 6. (B) Qualitative images of podocalyxin staining in thalamus, SNpr, and the entorhinal cortex in vehicle C57BL6 mice. (C) Percentage of marked area by TH-positive dendrites in medial and lateral SNpr 30 days post LPS injection. Medial SNpr: Unpaired t-test FTreatment 4.796, p < 0.3450. Lateral SNpr: Unpaired t-test FTreatment 2.171, p < 0.6308. (D) Percentage of marked area by Gephyrin-positive nerve terminals in medial and lateral SNpr 30 days post LPS injection. Medial SNpr: Unpaired t-test FTreatment 4.743, p < 0.3483. Lateral SNpr: Unpaired t-test FTreatment 4.349, p < 0.3739. (E) Representative images of Gephyrin-positive profiles in medial SNpr in Vehicle and LPS treated mice 30 days post-injection. [file Image_8.JPEG]

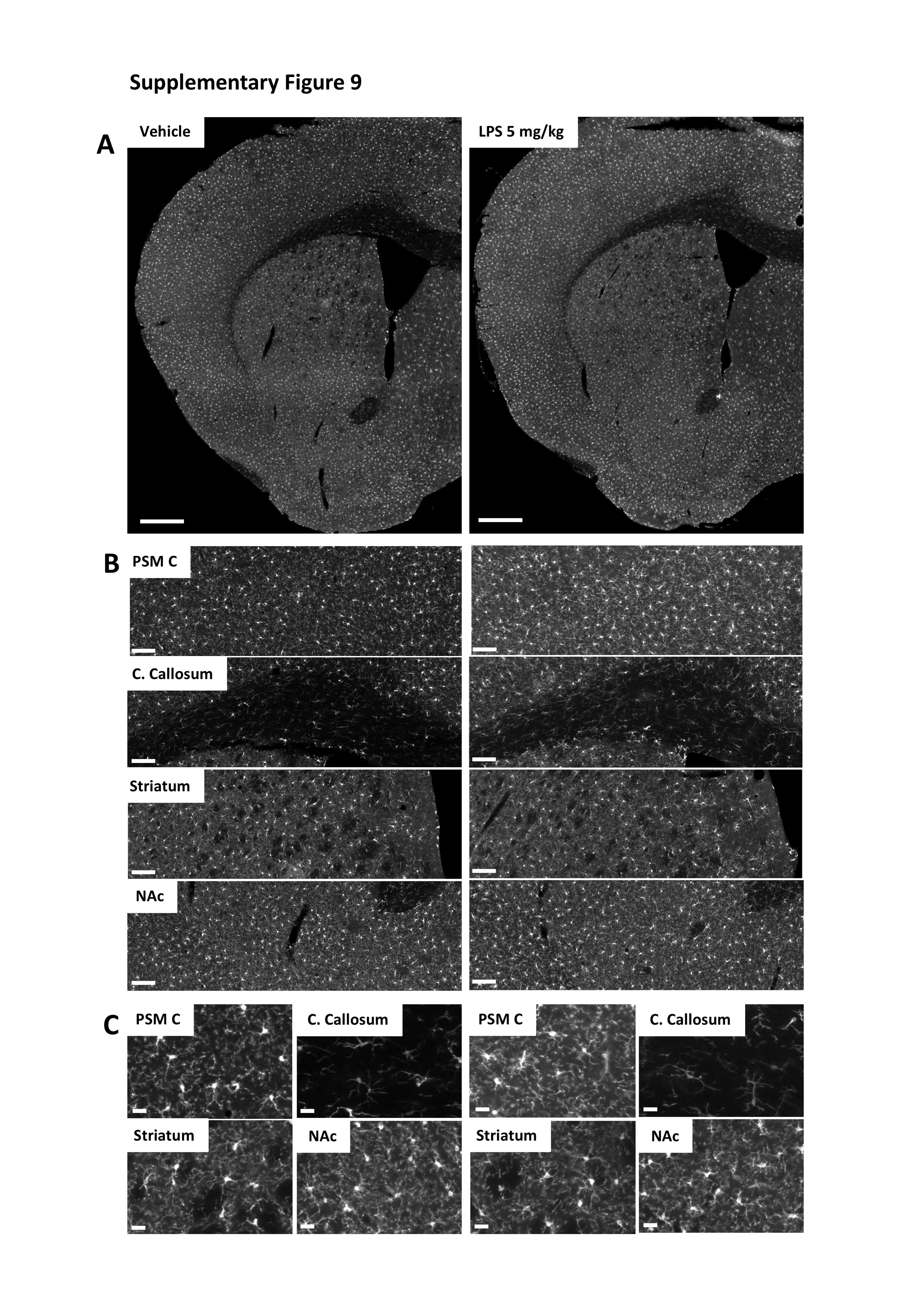

Supplement: Supplementary Figure 9 — Qualitative images of chronic inflammation in mice treated with 5 mg/kg and relative vehicles. This panel of images shows the different microglia activation 30 days after LPS administration in the PSM cortex with respect to all other regions in the same section. (A) Scale bars = 500 um. (B,C) High power images of PSM cortex, corpus callosum, striatum, and NAc in the same sections. (B) Scale bars = 100 um. (C) Scale bars = 20 um. [file Image_9.JPEG]

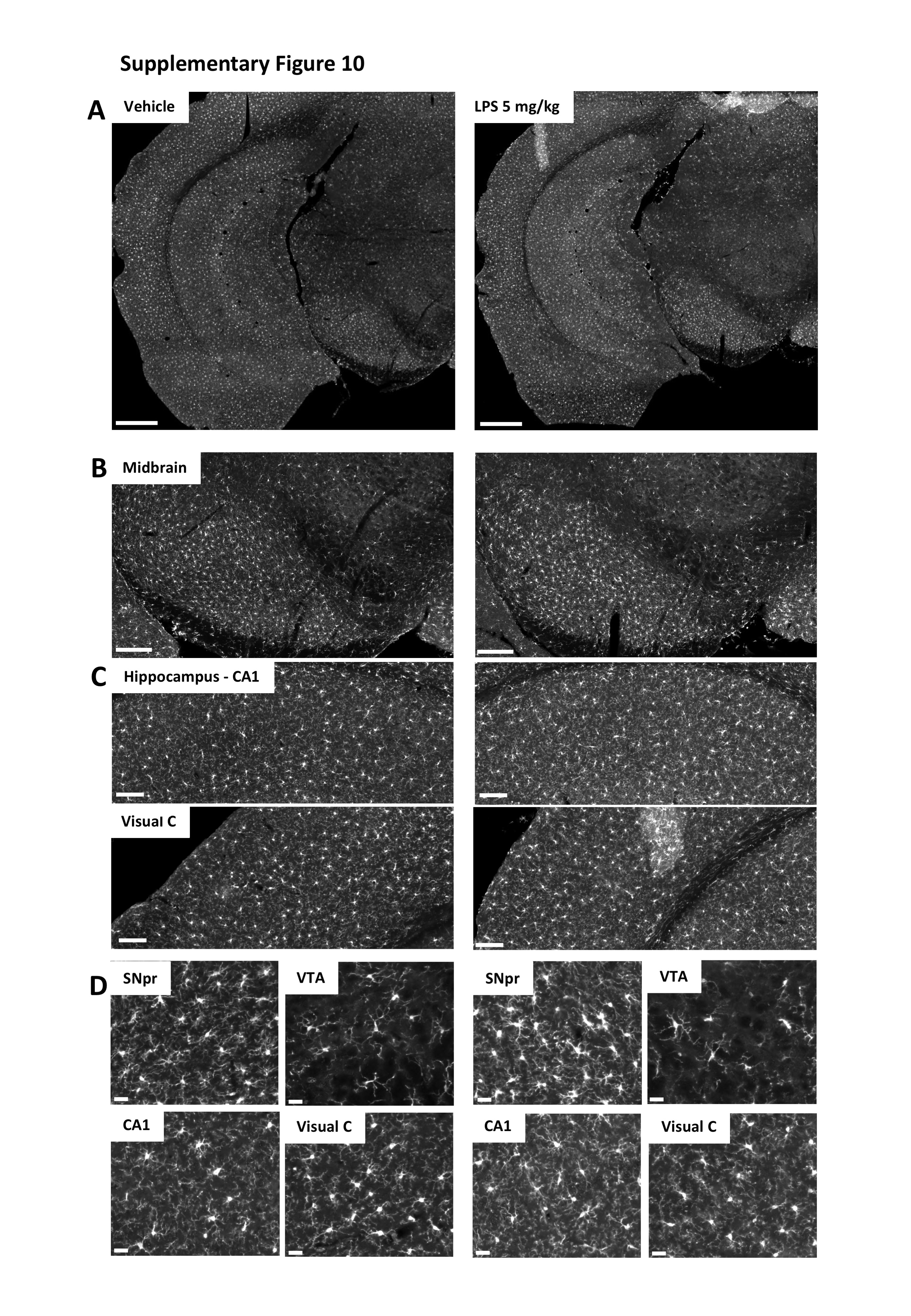

Supplement: Supplementary Figure 10 — Qualitative images of chronic inflammation in mice treated with 5 mg/kg and relative vehicles. This panel of images shows a different microglia activation 30 days after LPS administration in SNpr and hippocampus with respect to the VTA and visual cortex in the same section. (A) Scale bars = 500 um. (B) High power images of SNpr and VTA in the same sections. Scale bars = 200 um. (C) High power images of the hippocampus and visual cortex in the same sections. Scale bars = 100 um. (D) High power images of SNpr, VTA, CA1, and visual cortex in the same sections. Scale bar 20 um. [file Image_10.JPEG]

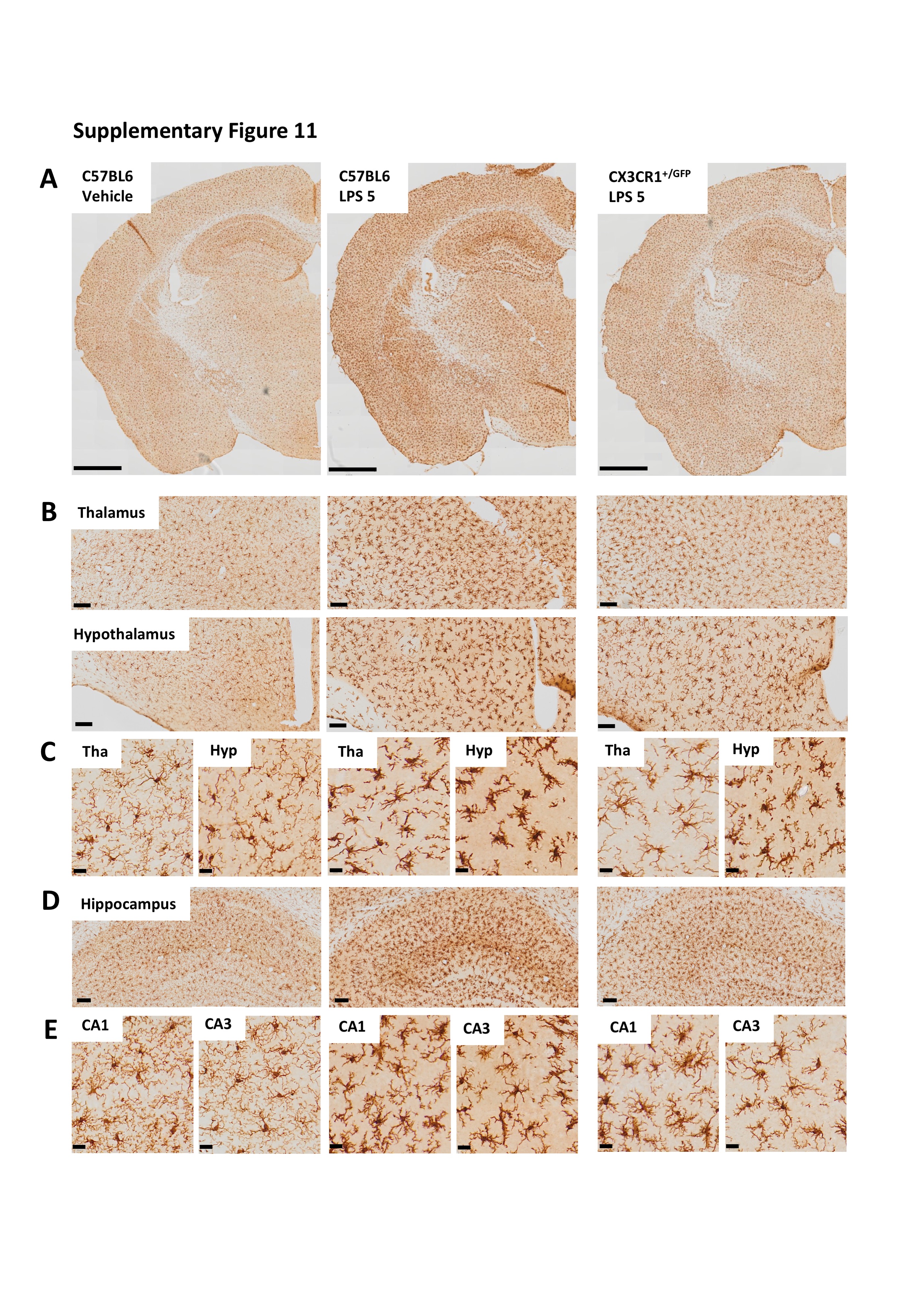

Supplement: Supplementary Figure 11 — Qualitative images of inflammation in C57BL6 and CX3CR1+/GFP mice treated with 5 mg/kg and relative vehicles for 24 h. This panel of images shows the lower microglia activation in CX3CR1+/GFP mice with respect to C57BL6 mice. (A) Scale bars = 800 um. (B,C) High power images of the thalamus and hypothalamus in the same sections. (B) Scale bars = 100 um. (C) Scale bars = 20 um. (D,E) High power images of hippocampus in the same sections. Scale bars = 100 um. (E) High power images CA1 and CA3 in the same sections. Scale bars = 20 um. [file Image_11.jpg]

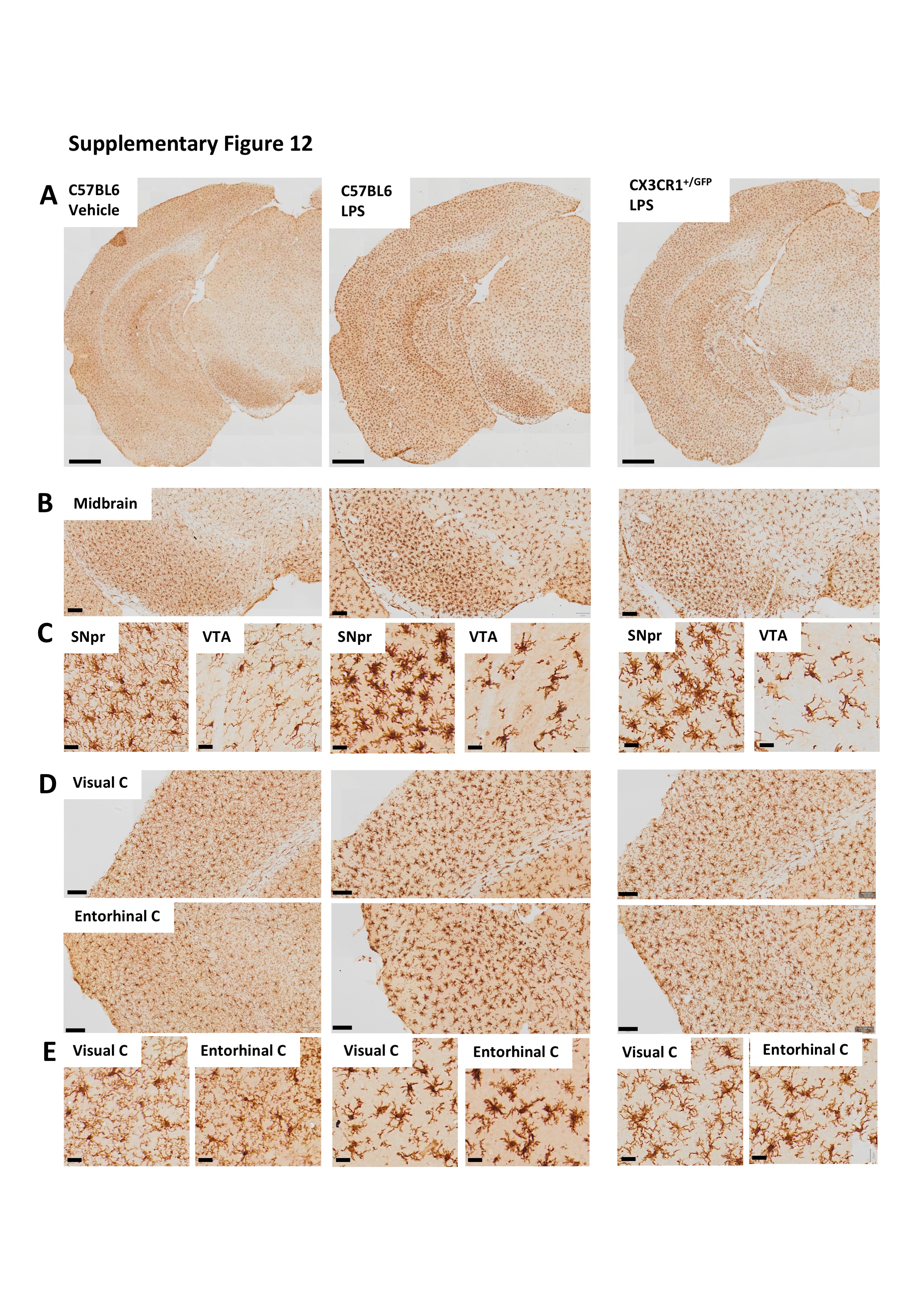

Supplement: Supplementary Figure 12 — Qualitative images of inflammation in C57BL6 and CX3CR1+/GFP mice treated with 5 mg/kg and relative vehicles for 24 h. This panel of images shows the lower microglia activation in CX3CR1-GFP mice with respect to C57BL6 mice. (A) Scale bars = 500 um. (B,C) High power images of SNpr and VTA in the same sections. (B) Scale bars = 100 um; (C) scale bars = 20 um. (D,E) High power images of visual cortex and entorhinal cortex in the same section. (D) Scale bar = 100 um. (E) Scale bars = 20 um. [file Image_12.jpg]

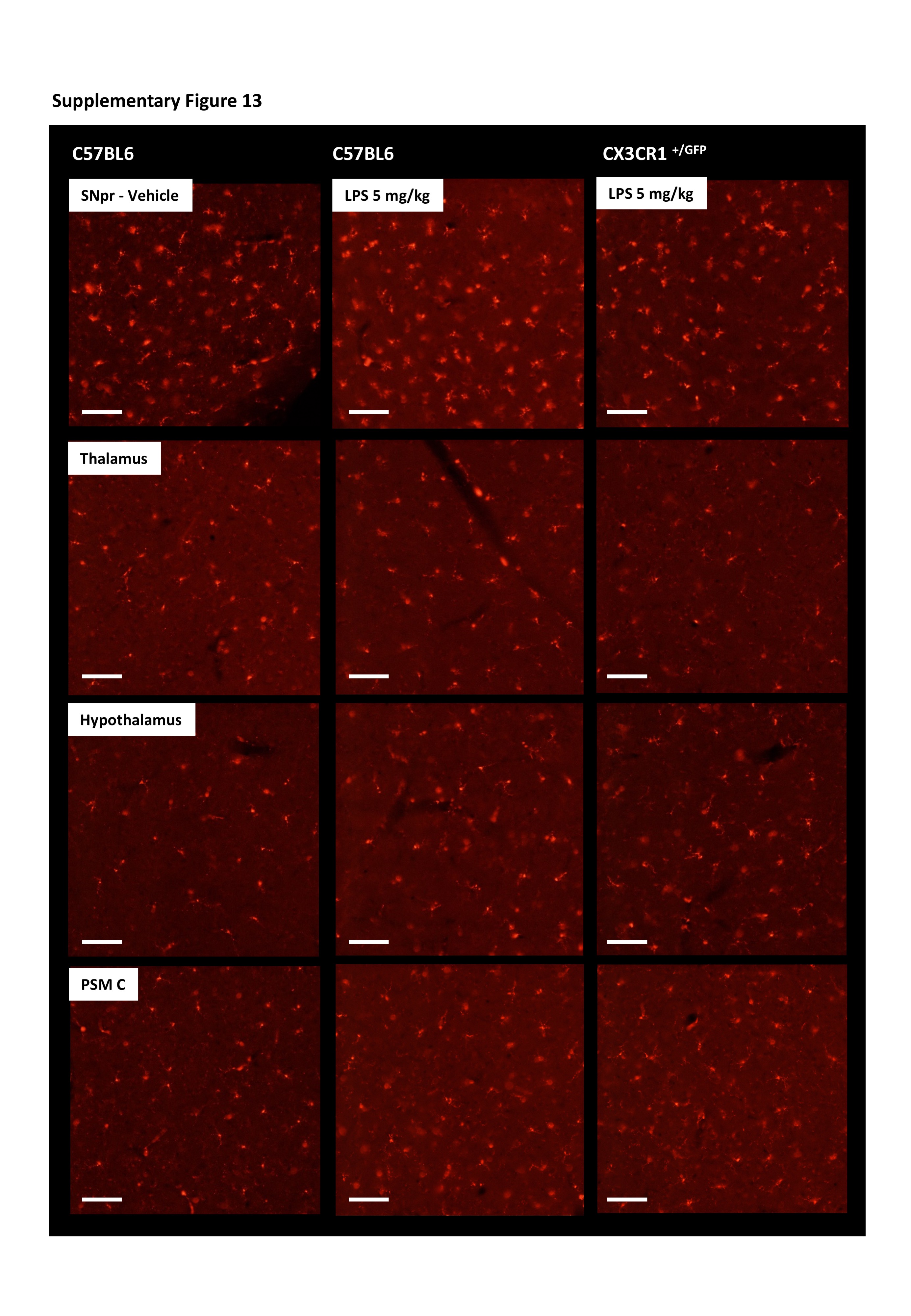

Supplement: Supplementary Figure 13 — Qualitative images of CD68 staining in C57BL6 and CX3CR1+/GFP treated with 5 mg/kg of LPS and relative vehicles. This panel compares the increase of CD68 in SNpr, thalamus, hypothalamus and PSM cortex. It is possible to appreciate the absence or lower activation state in CX3CR1+/GFP mice treated with LPS compared with the same group in C57BL6 mice. Scale bars = 40 um. [file Image_13.jpg]

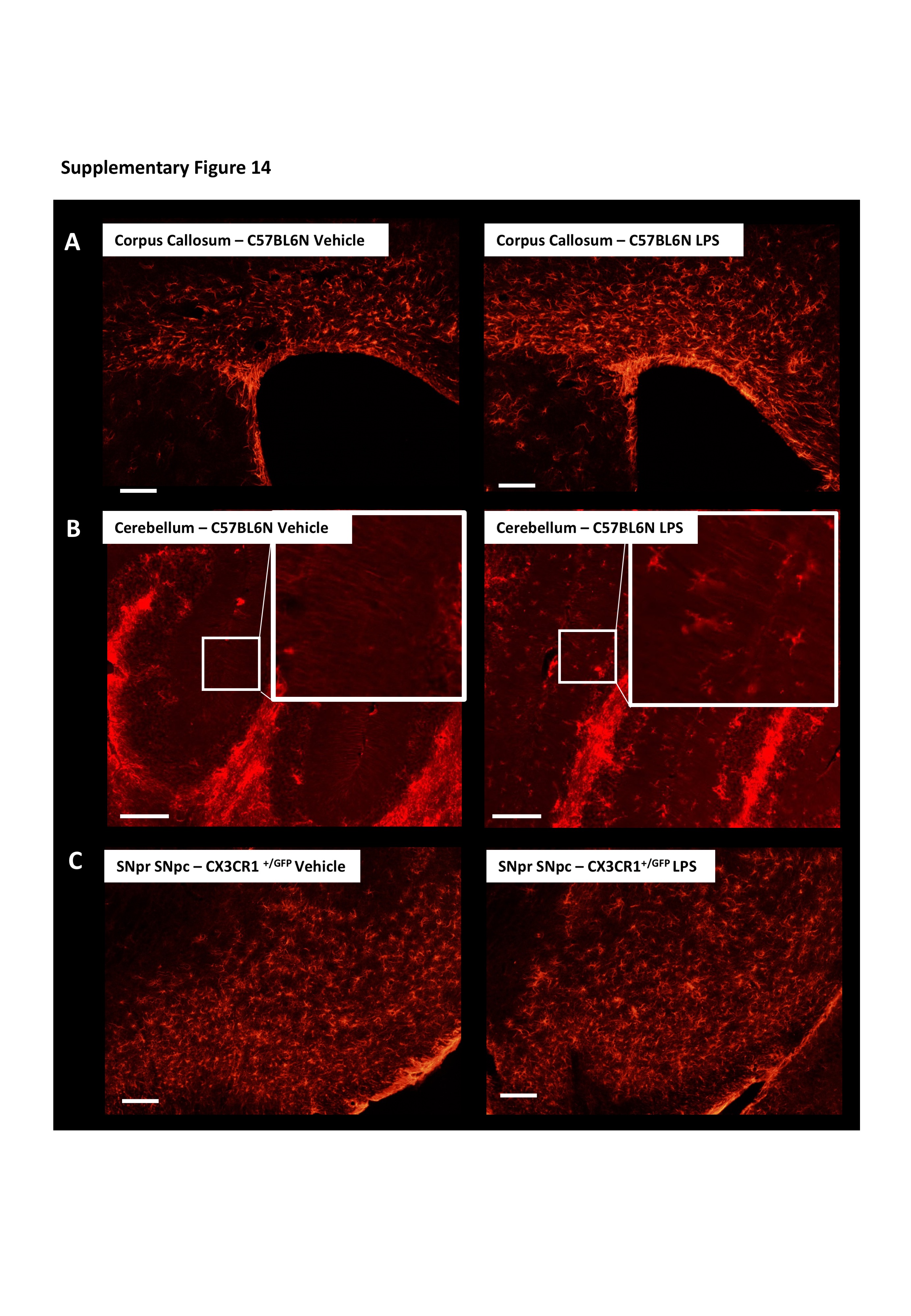

Supplement: Supplementary Figure 14 — (A) Qualitative images of GFAP staining in C57BL6 mice treated with 5 mg/kg of LPS and relative vehicles. It is possible to appreciate activation state in the corpus callosum of these mice. (B) Qualitative images of GFAP staining with different magnification in C57BL6 mice treated with 5 mg/kg of LPS and relative vehicles. The peculiar morphology observed in cerebellum with the appearance of astrocytes in the cortical region of the simple lobule. Particular examples were showed in the insets. (C) Qualitative images about the absence of astrocytic activation in SNpr in CX3CR1+/GFP mice treated with LPS and relative vehicle. [file Image_14.jpg]
